# Rebound increase in microRNA levels at the end of 5-FU-based therapy in colorectal cancer patients

**DOI:** 10.1038/s41598-023-41030-7

**Published:** 2023-08-30

**Authors:** Doaa Badr, Mariam A. Fouad, Marwa Hussein, Salem Salem, Abdelrahman Zekri, Samia Shouman

**Affiliations:** 1 Pharmacology and Experimental Oncology Unit, Cancer Biology Department, National Cancer Institute, Cairo University, Cairo, Egypt; 2 Cancer Signaling and Microenvironment Program, Fox Chase Cancer Center. 333 Cottman Avenue, Philadelphia, PA 19111 USA; 3 Medical Oncology Department, National Cancer Institute, Cairo University, Cairo, Egypt; 4 Virology and Immunology Unit, Cancer Biology Department, National Cancer Institute, Cairo University, Cairo, Egypt

**Keywords:** Cancer, Molecular medicine

## Abstract

Treatment with 5-fluorouracil (5-FU) based therapy is still used for colorectal cancer (CRC). Epigenetics has become a focus of study in cancer because of its reversibility besides its known regulatory functions. In this study, we will monitor the change in microRNAs (miRNAs) levels with 5-FU-based therapy at baseline and after 3 and 6 months of treatment to be correlated with their prognostic potential. The expression levels of 5 miRNAs, namely miRNA223-3p, miRNA20a-5p, miRNA17-5p, miRNA19a-3p, and miRNA7-5p, were measured in the peripheral blood of 77 CRC patients, along with the expression of 3 proteins PTEN, ERK, and EGFR. At baseline, CRC patients had significantly higher levels of circulating miRNAs than healthy controls. This level was reduced after 3 months of 5-FU-based therapy, then increased after 6 months significantly in responder patients compared to non-responders. MiRNA19a-3p showed that significant pattern of change in the subgroups of patients with high ERK, EGFR, and PTEN protein levels, and its 6 months level after 5-FU-based therapy showed significance for the hazard of increased risk of disease recurrence and progression.

## Introduction

Colorectal cancer (CRC) is the third most morbid cancer worldwide, according to the GLOBOCAN statistics, and the second mortality-causing type of cancer^1^. The treatment with 5-fluorouracil (5-FU) is still in use either in its prodrug form of oral capecitabine or as a drug injection received alone or in combination with other chemotherapeutics^2^. About 20–25% of CRC patients have metastatic disease at diagnosis, and it was found that 30% of patients would get a metastatic relapse following initial curative surgical treatment, with or without adjuvant chemotherapy^3^.

The small non-coding sequence of nucleotides, namely microRNAs (miRNAs), are known for their genomic transcriptional regulation activity to maintain genomic homeostasis. Some of these miRNAs become oncogenic and get up-regulated in cancer, and they are called oncomeres. These oncomeres cause inhibition to some important tumor suppressors^4^, suppression to the immune system^5^, and get recognized in certain subgroups of patients^6,7^. This inverse relation between oncomeres and the products of their target sits could be attributed to either reduced translational efficiency of the mRNA interaction site or an actual decrease in the transcript levels^8^. Other miRNAs act as tumor suppressors as miRNA15a and miRNA16-1 in chronic lymphocytic leukemia and let7 in the lung, breast, urothelial and cervical cancer^9^. Bioinformatics integration with large-scale miRNA array and sequencing revealed the up- and down-regulated miRNA in relation to the affected downstream signaling^10^. Their functional impact in CRC was described in their impact on Wnt/Notch/AKT/PI3K pathways leading to altered tumor growth and in TGF-β and EMT pathways promoting metastasis^8^.

MiRNAs have been identified as successful diagnostic markers for CRC in samples from blood^11^ and stool^12,13^. Also, their prognostic potential and response prediction in CRC were exhibited in both tissue^14,15^ and blood^6^. Plasma miRNA21, miRNA20a, and miRNA23a got significant discrimination power in CRC to identify the responders and the non-responders when combined with CEA and CA19.9^16^. Even alone, they possess the characteristics of biomarkers required to stratify different sub-groups of patients based on their microsatellite instability (MSI) status^17^. Also, CRC patients with positive lymphatic invasion have been presented to have aberrant expression of miRNA21 and miRNA135b^18^.

In a previous microRNA array screen conducted in the Egyptian National Cancer Institute, it was found that certain panel of blood circulating microRNAs (miRNA223-3p, miRNA20a-5p, miRNA17-5p, miRNA19a-3p, miRNA7-5p) have diagnostic potential in CRC^19^. Then we followed up in the current study by examining the prognostic potential of this panel prospectively. We monitored the change in miRNA levels with 5-FU-based therapy in relation to protein expression level and patients` response to therapy. We correlated the levels of these miRNAs with the levels of three proteins commonly associated with CRC progression: one tumor suppressor (PTEN) and two oncogenes (EGFR and ERK)^20^.

A number of 77 patients with confirmed diagnosis with CRC received 5-FU-based therapy for 6 months. During this period, 60 and 41 of them were sampled after 3 months and at the end of the treatment period, respectively. Patients were categorized into subgroups according to their clinicopathological features and followed up for disease recurrence and progression for 3 years.

An initial reduction in miRNA levels was found after 3 months of 5-FU-based therapy. Then a rebound increase in the levels of miRNAs was observed after 6 months of treatment to be in the same level or higher than the level of miRNAs at baseline. This fluctuating behavior of miRNAs was significant in responder patients compared to non-responders, and the change in miRNA19a-3p significantly associated CRC patients with high ERK, EGFR, and PTEN protein levels making the level of miRNA19a-3p at the end of 5-FU-based therapy a significant predictor for disease recurrence and progression.

## Patients and methods

### Patients

This is a prospective study with 77 pathologically proven CRC patients and 20 age and sex-matched healthy control individuals. All patients had normal organ functions, and their demographics were recorded. All patients were chemo naive and received 5-FU-based therapy in doses and duration described in the protocols of CRC treatment followed by the Medical Oncologists in the Egyptian National Cancer Institute, Cairo University. At baseline, 77 patients were approved to participate in the study. Peripheral blood samples were collected from all patients and control groups to measure peripheral miRNA and protein levels. From those 77 CRC patients, 60 and 41 patients were sampled again after 3 and 6 months of 5-FU-based therapy.

In patients with stage II disease with high-risk features, adjuvant capecitabine was administered orally as a single agent in a 1,250 mg/m2 dose twice/day for two weeks, followed by a 1-week rest period, given as 3-week cycles for a total of eight cycles. While stage III and metastatic patients received XELOX in 3-week treatment cycles in which intravenous oxaliplatin 130 mg/m2 was given on day 1, followed by oral capecitabine 1,000 mg/m2 twice daily for two weeks and a one-week rest.

The study was approved by the Institutional Human Research Ethics Committee of the Egyptian National Cancer Institute, Cairo University, Number 00004025, with IRB review Number 2010014019.3. Written informed consent was obtained from all participants for using their blood samples in this study. Patients were followed for 3 years till the primary endpoint of event-free survival (EFS), calculated from the date of primary treatment till the date of relapse or progressive disease, and the secondary end-point of overall survival (OS) calculated from the date of diagnosis till the date of death. Living patients or patients lost to follow-up were censored on the last known alive date.

### Serum samples collection

A whole peripheral blood sample (5 ml) was collected in an anti-coagulant-free Vacuette® blood collection tube (Greiner Bio-One, Kremsmünster, Austria) to isolate serum before the start of treatment (baseline) and then after 3 and 6 months of 5-FU-based therapy. The blood serum was separated by centrifugation at 1400 rpm for 10 min, and the clear serum supernatant was stored in RNase-free Eppendorf tubes at -80 °C for further use.

### RNA extraction and qRT-PCR

Total RNA, including miRNAs, was extracted from 200µL of thawed serum using the miRNeasy Mini Kit (Qiagen, Valencia, CA, USA, Cat. No. 217004) according to the manufacturer's instructions. The RNA content (ng/µl) was measured using the NanoDropTM (Thermo Fisher Scientific, USA). According to the manufacturer's protocol, cDNA Synthesis was performed using miScript II RT kit (Qiagen, Valencia, CA, USA, Cat. No. 218161) on 100 ng of total RNA in a final volume of 20 µl. Quantitative real-time PCR (qRT-PCR) was performed using miScript SYBRÒ Green PCR kit (Qiagen, Valencia, CA, USA, Cat. No. 218075). Mature miRNAs expression levels were generated in 96-well arrays using the custom miScript miRNA RT-PCR for miRNA223-3p, miRNA20a-5p, miRNA17-5p, miRNA19a-3p, miRNA7-5p (Qiagen, Valencia, CA, USA, Cat. No. CM1HS0064C) according to the provided instructions as follows: 15 min at 95 °C for 1 cycle, 15 s at 94 °C, 30 s at 55 °C, and 34 s at 70 °C for 45 cycles using AB7500 Fast Real-Time PCR system. Threshold cycle data were analyzed using the RT2 Profiler Software (Version 3.4; SABiosciences). To obtain serum levels of studied miRNAs, relative gene expression levels were normalized to those obtained from the amplification of RNU6, and the relative quantity of miRNA was calculated according to 2^-**ΔΔCT**^ and log2^-**ΔΔCT**^ method.

### ERK, EGFR and PTEN protein levels.

The ERK, EGFR, and PTEN protein concentration was determined using ELISA kits (Sunlong Biotech Co., LTD, Hangzhou, Zhejiang, China). Procedures were carried out following the manufacturer's instructions. The concentration of the markers in plasma samples was calculated by comparing the samples' optical density (OD) to the corresponding plotted standard curves.

### Statistical analysis

Data management and analysis were done using IBM SPSS Statistics for Windows version 24.0 SPSS Inc., Chicago, IL, USA. The Kolmogorov–Smirnov and Levene's tests assessed the normal distribution and variance homogeneity of data. Numerical data were summarized using median and interquartile range (IQR). Categorical data were summarized as counts and percentages. Comparison of miRNAs between patients and the control group, or between the subgroups of patients, were done using the Mann–Whitney test (when comparing two independent groups) and Kruskal–Wallis (when comparing more than two independent groups). The change in the miRNAs and proteins levels under the effect of 5-FU-based therapy over time was tested for significance with Friedman test when comparing more than two dependent groups, and Wilcoxon matched test when comparing two dependent groups only. Associations between categorical variables were performed using Pearson's chi-square. Spearman's test was used to detect the strength of the correlation between the tested markers. For diagnostic sensitivity and specificity evaluation, receiver operating characteristic (ROC) curves were constructed, and the areas under the curves (AUC) were estimated. The cumulative survival rate, and median levels of OS and EFS, were estimated with Kaplan–Meier method and log-rank test for subgroups` survival curves comparisons. The hazard of disease recurrence and progression was estimated using Cox Proportion Hazard Model for the levels of miRNAs at baseline and after 3 and 6 months of 5-FU-based therapy. *P*-values are two-sided and were considered significant at 0.05 levels in all analyses except in subgroup analysis; the significance level decreased to 0.01.

### Institutional review board statement

The study was conducted in accordance with the Declaration of Helsinki and approved by the Institutional Human Research Ethics Committee of the Egyptian National Cancer Institute, Cairo, Egypt, Number 00004025, with IRB review Number 2010014019.3.

### Informed consent statement

Informed consent was obtained from all subjects involved in the study.

## Results

### CRC patients' clinicopathological characteristics

Table S1 shows the demographics of 77 CRC patients accepted to participate in this study. At baseline, the median age of this group of patients was 47 years. The male/female ratio was 1:0.93. Most patients had good performance status according to ECOG classification (83.1%), and with positive family history in 16.9%. Colon and rectal cancers were diagnosed in 45 and 32 patients, respectively. Adenocarcinoma was the dominant pathological type, with histological variants of mucinous, signet ring and neuroendocrine in 16, 11 and 1 patients respectively. Most patients had grade II tumors [59 (67%)]. According to TNM classification, T3 was identified in 33 patients (43%), positive nodal disease was presented in 27 patients (35%), and 21 patients had metastatic disease. Surgical intervention was performed on 55 patients. All patients received chemotherapy, 5-FU-based, either in the adjuvant or metastatic settings, alone or in combination with other chemotherapeutic agents. At the end of therapy, 54.5% of patients were in complete remission. Baseline patients were monitored during their journey of treatment with 5-FU-based therapy. 60 and 41 of them were sampled after 3 and 6 months, respectively, however the count and % of patients remained insignificant among all subgroups.

### Significant elevation in the baseline levels of circulating miRNAs in 77 CRC patients amplifying their diagnostic features

**Figure 1:**
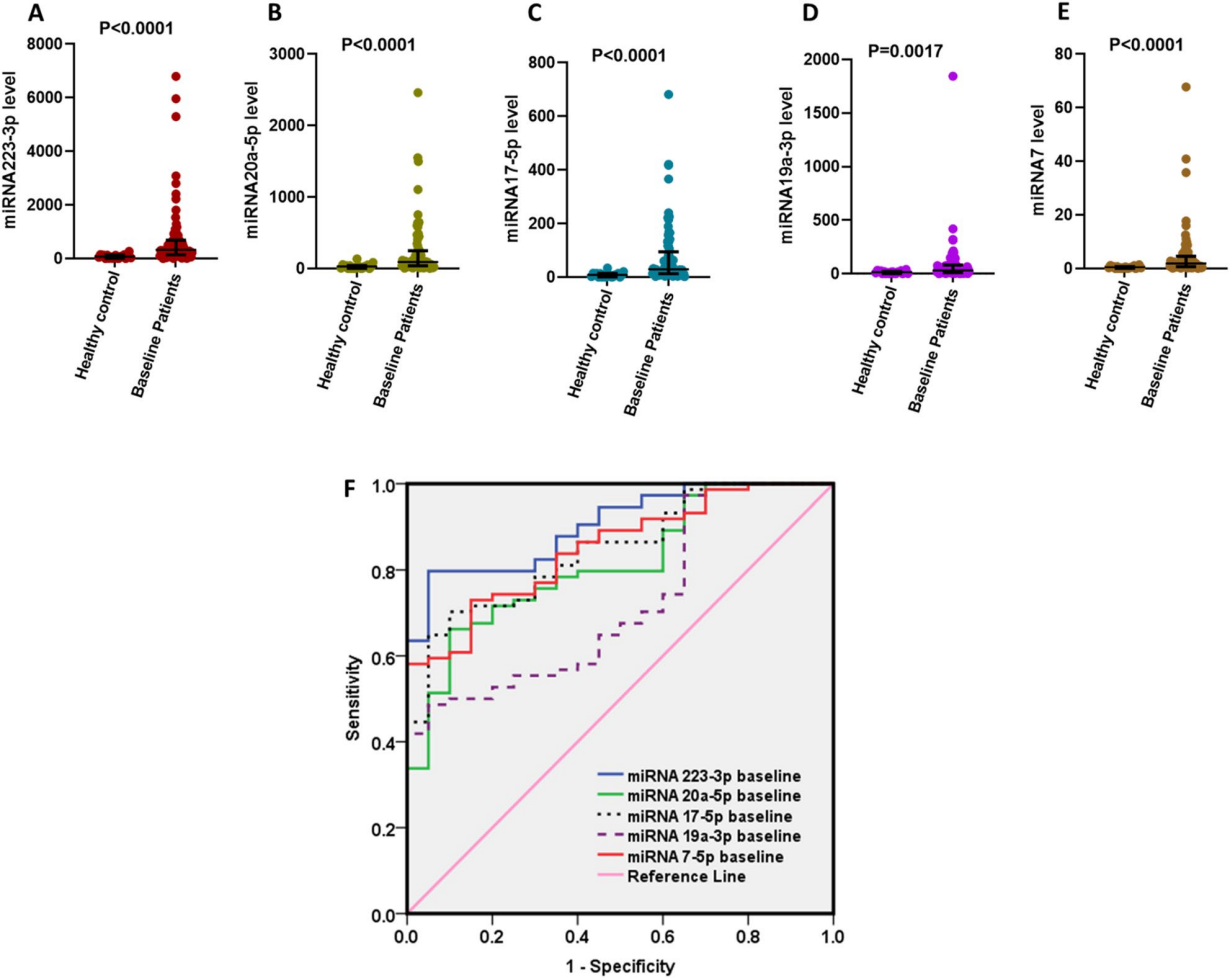
Expression levels of 2^−**ΔCT**^ of miRNAs in the serum of baseline 77 CRC patients compared to 20 healthy subjects. (**A**) miRNA223-3p, (**B**) miRNA20a-5p, (**C**) miRNA17-5p, (**D**) miRNA19a-3p, (**E**) miRNA7-5p, (**F**) ROC curve showing AUC of miRNAs at baseline patients compared to the line of reference. miRNAs levels are expressed as 2^−**ΔCT**^ relative to RNU6 amplification level.

Higher serum expression levels of miRNA223-3p, miRNA20a-5p, miRNA17-5p, miRNA19a-3p, and miRNA7-5p in the 77 CRC patients compared to the 20 healthy controls. The median (IQR) values of miRNA233-3p, miRNA20-5p, miRNA17-5p, miRNA19a-3p, and miRNA7-5p were 330.7 (144.7–692.7), 91.65 (38.86–251.0), 29.82 (13.02–94.74), 30.99 (10.18–80.82) and 1.93 (0.68–4.62) respectively versus their levels in healthy control which were 71.7 (10.47–131.4), 28.65 (3.01–41.36), 8.17 (0.85–14.13), 14.68 (0.60–20.69), 0.43 (0.11–0.72), Fig. 1A–E respectively.

In Fig. 1F, the expressions of all mentioned miRNAs were tested as diagnostic markers to differentiate between normal healthy individuals and CRC patients. Results of the used miRNAs showed high sensitivity and specificity for miRNA 223-3p (81% sensitivity and 90% specificity); miRNA20a-5p (74% sensitivity and 70% specificity); miRNA17-5p (70% sensitivity and 90% specificity); miRNA19a-3p, (50% sensitivity and 90% specificity) and miRNA7-5p (74% sensitivity and 80% specificity) with an area under the ROC curve of 0.907, 0.815, 0.847, 0.724 and 0.850 respectively.

### Paradoxically increased levels of miRNAs at the 6 months of treatment with 5-FU-based therapy, after initial reduction at the 3 months

After 3 months of 5-FU-based therapy, all studied miRNAs were down-regulated in CRC patients (n = 60) compared to their expression levels before the start of therapy. A rebound increase in the expression level of all miRNAs was observed after 6 months of therapy (n = 41). This trend was significant with miRNA223-3p, miRNA19a-3p, and miRNA7-5p (*P* = 0.002, 0.001, and 0.048, respectively). While the fluctuating trend was insignificant with miRNA20a-5p, miRNA17-5p, and the 3 measured proteins ERK, EGFR, and PTEN (Fig. S1). Spearman correlation did not show significance between all circulating miRNAs and the 3 proteins, just a weak inverse relation was exhibited after 3 months of 5-FU therapy clearly observed in metastatic patients (Fig. S2).

### Responder patients showed significant trend of reduction then increase in their miRNA levels over the treatment period with 5-FU-based therapy, and that change in miRNA19a-3p level was significant in patients with high basal protein levels

Doing inter-treatment comparison by stratifying the patients according to the treatment regimen either single 5-FU versus combination of 5-FU with oxaliplatin, showed significance in the trend of initial reduction then increase with the levels of miRNA223-3p (*P* = 0.008), with preferential significance toward the oxaliplatin combination (Table S2). However, we noticed a drop in the levels of all microRNAs in the subgroup of patients receiving oxaliplatin either at baseline (while assigning the patients to the appropriate protocol) or during the treatment cycles.

**Figure 2:**
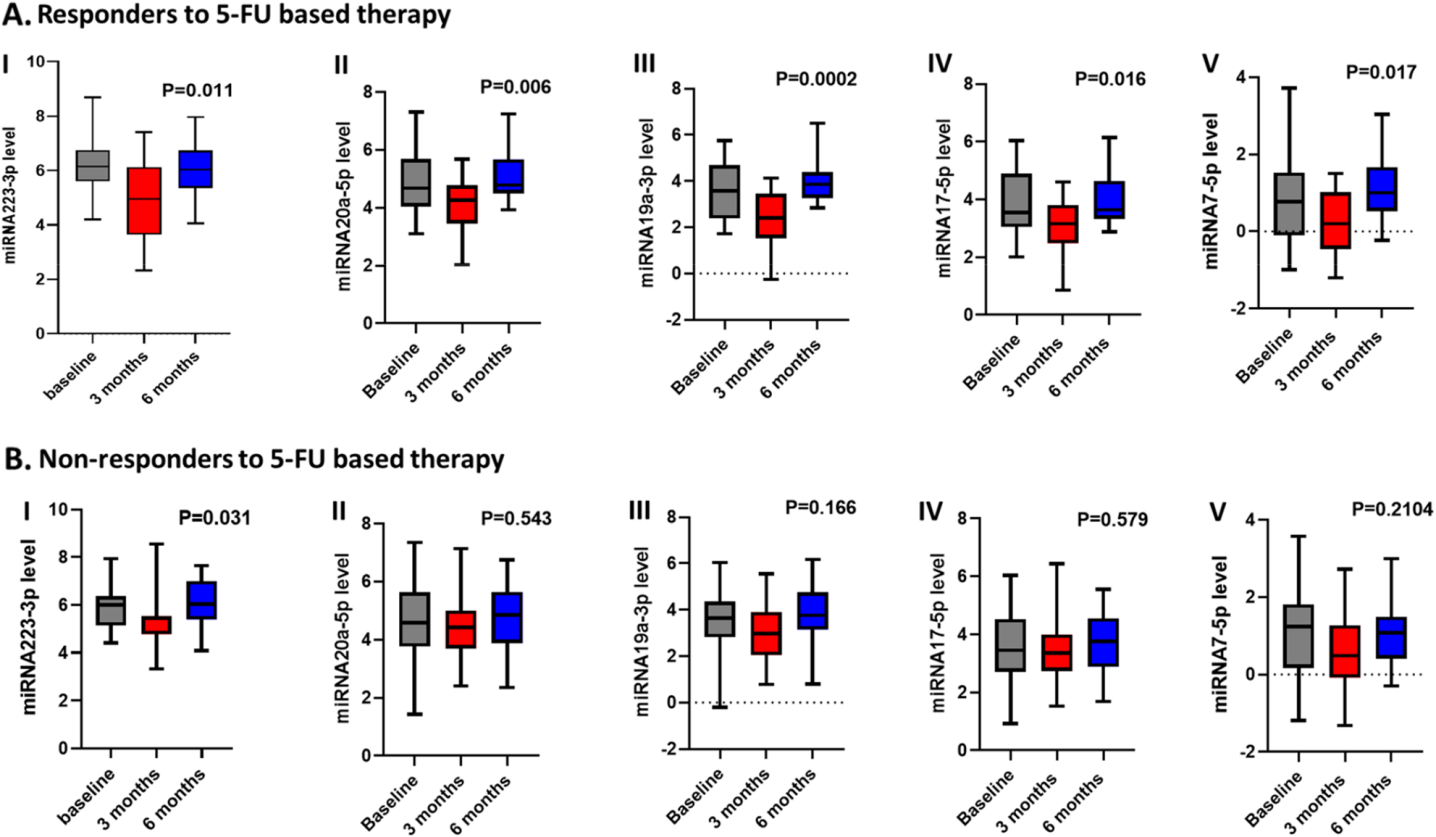
Change in miRNAs levels with 5-FU-based therapy in CRC patients at baseline, and after 3 and 6 months of treatment. (**A**) in responders (n = 23), **(B**) in non-responders (n = 20). **I.** miRNA223-3p, **II.** miRNA20a.5p, **III.** miRNA17-5p, **IV.** miRNA19a-3P, **V.** miRNA7-5p. The levels of miRNAs are expressed as log2^−**ΔΔCT**^**,** and *P*-values are displayed in the figures.

Responder patients tend to show a significant trend of change in miRNA levels than non-responders over the treatment period. The associated changes in miRNA levels with 5-FU-based-therapy in responder patients were significant by *P*-values of 0.011, 0.006, 0.0002, 0.016, and 0.017 compared to the P values documented with non-responders of 0.031, 0.543, 0.166, 0.579, and 0.2104 with miRNA223-3p, miRNA19a-3p, miRNA17-5p, miRNA7-5p, respectively, Fig. 2.

**Figure 3:**
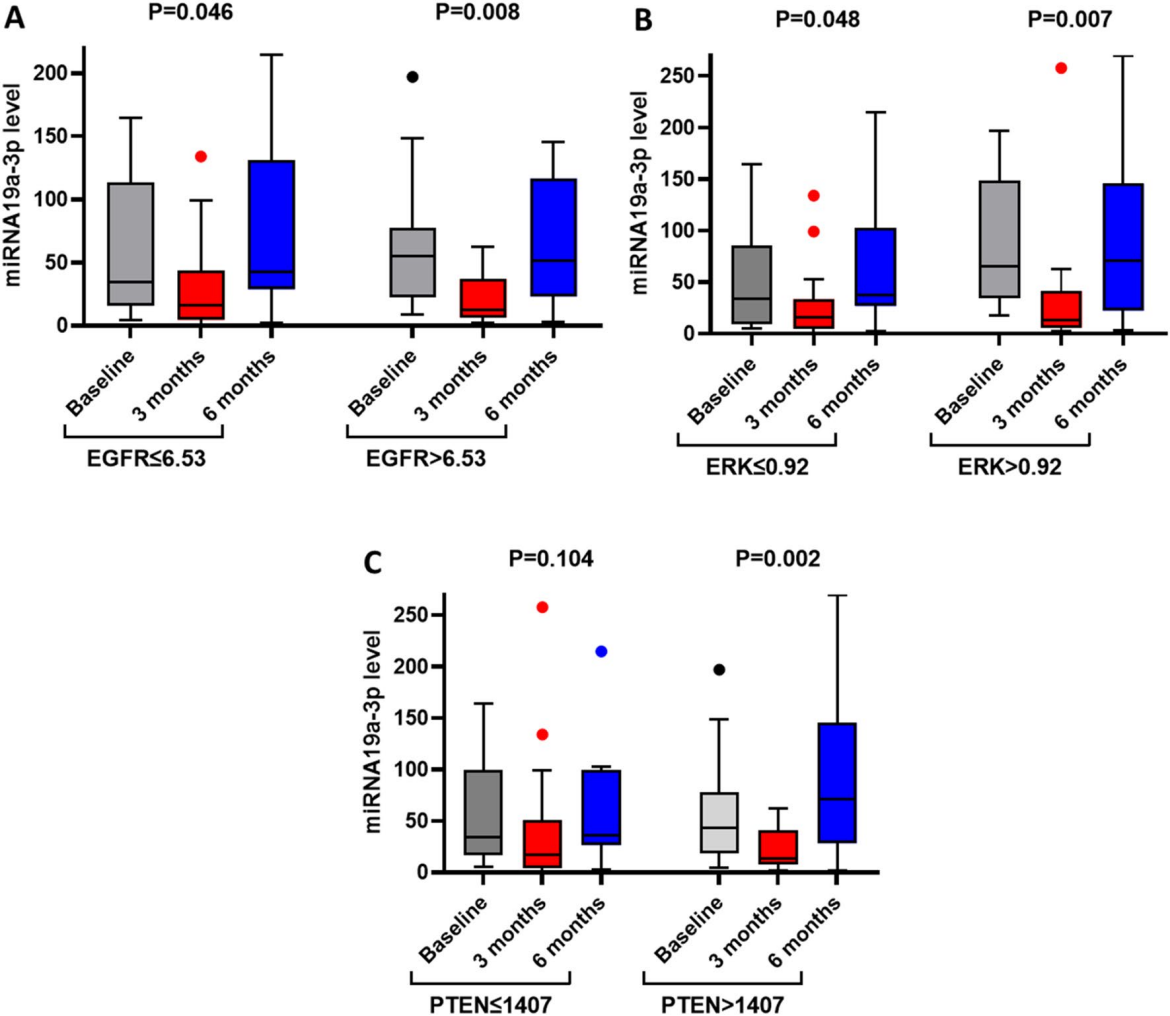
Change in miRNA19a-3p level in response to three and six months of 5-FU therapy in patients with low and high basal levels of proteins. (**A**) EGFR, (**B**) ERK (**C**) PTEN. The levels of miRNAs are expressed as log2^−**ΔΔCT**^**,** and the *P*-values are displayed in the figures.

Patients with basal levels of EGFR > 6.53, ERK > 0.92, and PTEN > 1407 showed a significant reduction of miRNA19a-3p at 3 months and then increase at the end of treatment compared to patients with basal protein levels equal or lower than the median values of these proteins (*P* = 0.008, 007 and 0.002 for patients with the high level of proteins compared to P = 0.046, 0.048 and 0.042 for patients with the low level of proteins, Fig. 3).

Adding to the above, there were multiple significances in that trend of rebound change in miRNA levels over the treatment period associated certain subgroups of patients. Significant rebound elevation in the expression of miRNA223-3p at the end of 5-FU-based therapy was observed in male patients (*P* < 0.001), smokers (*P* = 0.004), with colonic site disease (*P* = 0.002), patients with T3 tumors (*P* < 0.001), and those with positive lymph nodes (*P* = 0.001), Table S3. The re-bound increase of miRNA19a-3p in Table S5 was significant in male patients and those with colonic tumor (*P* = 0.003 and 0.004, respectively), while miRNA7-5p showed rebound significance in young patients (age ≤ 47 years, *P* = 0.006, Table S7).

### Significant high miRNA levels in the non-metastatic subgroup of patients at the 3 months sampling time

**Figure 4:**
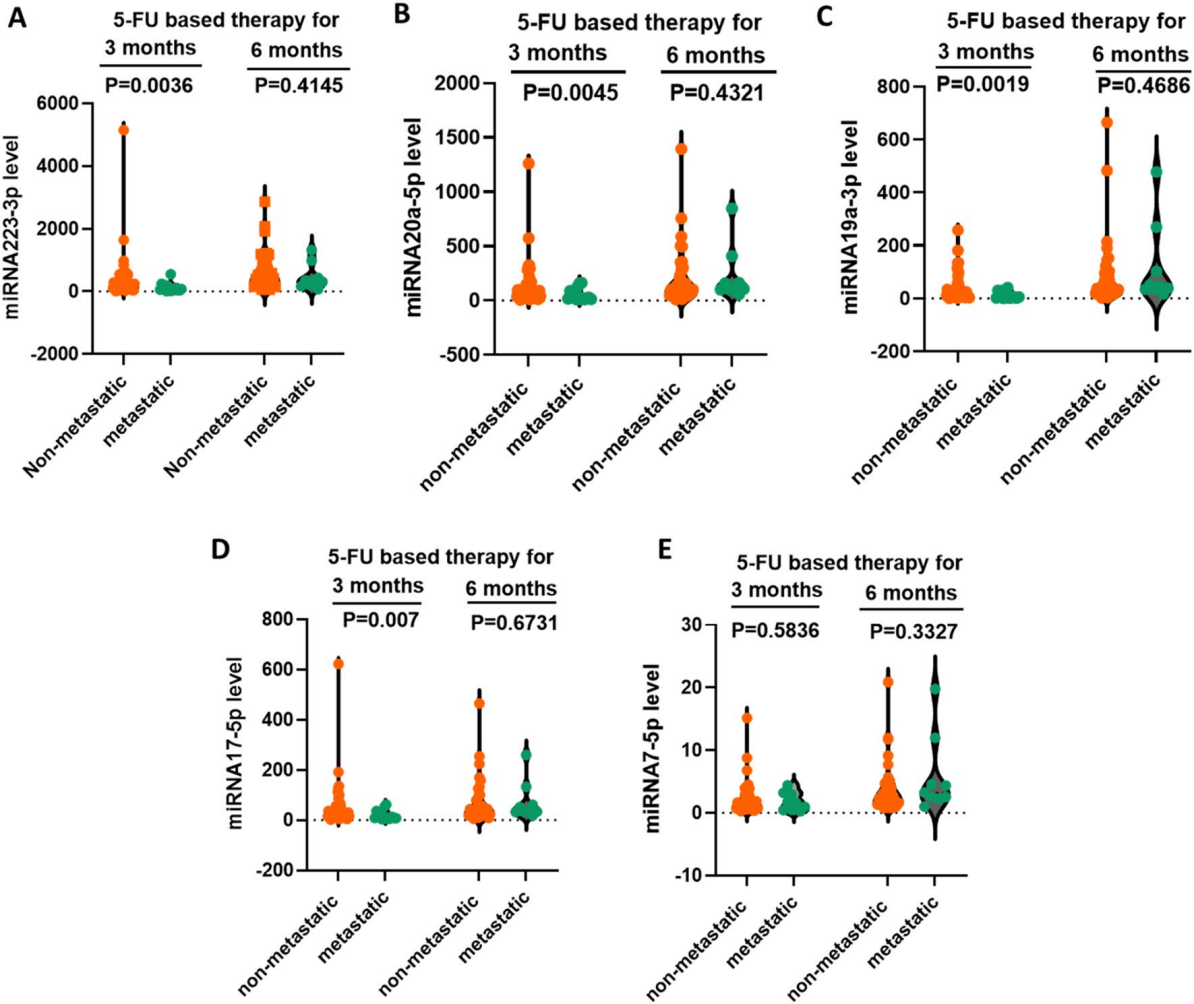
Level of miRNAs in non-metastatic and metastatic patients after three and six months of 5-FU therapy. (**A**) miRNA223-3p, (**B**) miRNA20a-5p, (**C**) miRNA19a-3p, (**D**) miRNA17-5p, (**E**) miRNA7-5p. The levels of miRNAs are expressed as log2^−**ΔΔCT**^ and *P*-values are displayed on the figures.

Non-metastatic patients have significantly higher miRNA levels after 3 months of 5-FU-based therapy than metastatic patients. After 3 months of 5-FU therapy, all miRNAs, except miRNA7-5p, showed significantly higher levels of expression in non-metastatic patients than metastatic ones (*P* = 0.0036, 0.0045, 0.0019, 0.007, and 0.0.584 for miRNA223-3p, miRNA20a-5p, miRNA19a-3p, miRNA17-5p and miRNA7-5p, respectively). However, after 6 months of 5-FU-based therapy, the difference in the miRNA expression levels become insignificant between the non-metastatic and metastatic patients, Fig. 4.

Separately, by measuring the levels of miRNAs at this time point of 3 months, we found significant association between high miRNA17-5p and miRNA7-5p with low ERK protein level (Table 1). Also, miRNA17-5p level was significantly high with low EGFR, but high PTEN protein levels (Table 1).

**Table 1:** Change in miRNA levels with low and high EGFR, ERK, and PTEN protein levels.

Subgroups		Baseline			3 months of 5-FU therapy			6 months of 5-FU therapy			*P*-value
*miRNA223-3p*											
ERK	≤ 0.92	6.22	5.65	6.39	5.52	4.24	6.12	5.82	5.39	6.39	0.247
	> 0.92	6.07	5.77	6.76	5.2	4.65	5.82	6.32	5.34	7.08	0.031
*P*-value		0.121			0.895			0.303			
PTEN	≤ 1407.85	6.28	5.94	6.57	5.52	3.51	6.41	5.82	5.09	6.39	0.344
	> 1407.85	6.07	5.65	6.39	5.38	4.65	5.55	6.32	5.52	7.08	0.011
*P*-value		0.119			0.885			0.228			
EGFR	≤ 6.53	6.28	5.74	6.57	5.49	4.24	6.41	6	5.39	6.89	0.247
	> 6.53	6.19	5.81	6.55	5.2	4.65	5.82	6.05	5.34	6.71	0.038
*P*-value		0.12			0.728			0.915			
*miRNA20a-5p*											
ERK	≤ 0.92	4.74	4.19	6.01	4.29	3.47	5.13	4.62	4.05	5.43	0.766
	> 0.92	4.66	4.34	5.22	4.42	3.6	4.74	5.07	4.52	6.21	0.155
*P*-value		0.121			0.057			0.707			0.364
PTEN	≤ 1407.85	4.52	4.1	6.01	4.42	3.47	5.13	4.62	4.13	5.61	0.936
	> 1407.85	4.87	4.59	5.77	4.3	3.6	4.74	5.07	4.05	6.02	0.085
*P*-value		0.119			0.036			0.675			0.812
EGFR	≤ 6.53	4.74	4.19	6.01	4.29	3.47	5.13	4.62	4.05	5.43	0.766
	> 6.53	4.66	4.34	5.22	4.42	3.6	4.74	5.07	4.52	6.21	0.155
*P*-value		0.12			0.057			0.707			0.364
*miRNA19a-3p*											
ERK	≤ 0.92	3.53	2.23	4.45	2.78	1.57	3.52	3.63	3.29	4.63	0.165
	> 0.92	4.18	3.54	5	2.58	1.76	3.72	4.26	3.1	4.98	***0.007***
*P*-value		0.121			0.018			0.757			0.669
PTEN	≤ 1407.85	3.53	2.82	4.6	2.81	1.43	3.92	3.59	3.27	4.6	0.42
	> 1407.85	3.77	2.91	4.35	2.58	2.06	3.72	4.26	3.34	4.98	***0.005***
*P*-value		0.119			0.101						0.73
EGFR	≤ 6.53	3.53	2.84	4.6	2.81	1.55	3.92	3.63	3.29	4.63	0.42
	> 6.53	3.87	2.91	4.35	2.47	1.8	3.17	3.94	3.24	4.8	***0.005***
*P*-value		0.12			0.09			0.696			0.768
*miRNA17-5p*											
ERK	≤ 0.92	3.3	3.09	3.89	3.37	2.72	4.19	3.49	3.09	4.38	0.819
	> 0.92	4.15	3.61	4.89	3.31	2.47	3.76	3.95	2.9	5.06	0.041
*P*-value		0.121			***0.009***			0.646			0.448
PTEN	≤ 1407.85	3.62	3.11	4.89	3.16	2.49	4.19	3.46	2.97	4.38	0.936
	> 1407.85	3.7	3.29	4.66	3.41	2.66	3.59	3.95	3.51	5.06	0.127
*P*-value		0.119			0.062			0.744			0.212
EGFR	≤ 6.53	3.35	3.11	4.89	3.37	2.49	4.19	3.49	3.09	4.58	1
	> 6.53	3.74	3.48	4.76	3.31	2.66	3.59	3.95	2.97	4.89	0.074
*P*-value		0.12			0.043			0.679			0.647
*miRNA7-5p*											
ERK	≤ 0.92	1.23	− 0.17	1.73	0.61	− 0.23	1.38	0.55	0.44	1.48	1
	> 0.92	1.25	0.94	2.16	0.42	− 0.34	1.01	1.31	0.84	2.46	0.011
*P*-value		0.121			**< *0.001***			0.435			0.277
PTEN	≤ 1407.85	1.45	− 0.09	2.2	0.13	− 0.45	1.38	1.17	0.44	1.48	0.549
	> 1407.85	1.21	0.92	1.91	0.47	0.06	1.09	1.08	0.74	2.48	0.155
*P*-value		0.119			***0.002***			0.628			0.49
EGFR	≤ 6.53	1.41	− 0.09	2.2	0.47	− 0.34	1.36	1.19	0.44	1.64	0.766
	> 6.53	1.23	0.92	1.91	0.43	− 0.01	1.01	1.08	0.74	2.21	0.038
*P*-value		0.12			***0.001***			0.595			0.736

Data presented as medians and IQR of miRNAs level. Significant *P*-values ≤ 0.01 are displayed in bold- italic font.

*IQR* interquartile range.

### The level of miRNA19a-3p after 6 months of 5-FU therapy was significant predictor for the hazard of disease recurrence and progression

Tables S8 and S9 show the change in miRNA levels, either by increase or decrease at the end of 5-FU-baed therapy relative to their baseline expression level. Of the 77 patients, 18 males experienced an increase in miRNA223-3p at the end of therapy (*P* = 0.009). A significant percentage of patients who got increased levels of miRNA20a-5p and miRNA7-5p were diagnosed with T3 tumors, *P* = 0.006 and 0.002, respectively.

After 3 years of follow-up, patients` OS and EFS were estimated in the subgroups of CRC patients with low and high basal expression levels of miRNAs (Supplementary Figs. S3–S12). Generally, better survival rates were associated CRC patients with a high expression level of miRNAs before treatment, but the difference has not reached significance in all miRNAs.

**Figure 5:**
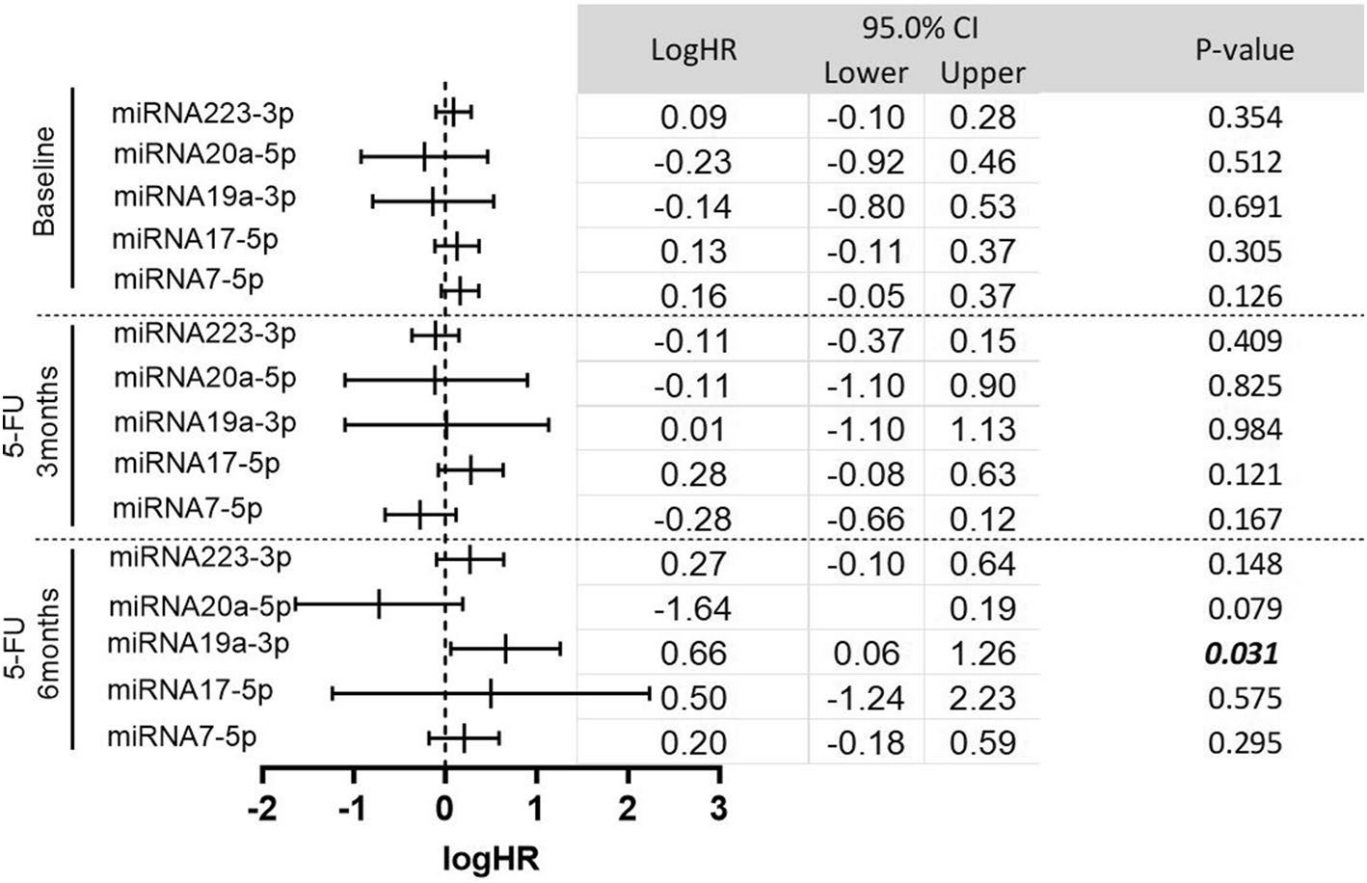
The hazard of disease recurrence and progression after 3 years of follow-up for 77, 60 and 41 CRC patients at baseline, after 3 months of 5-FU therapy, after 6 months of 5-FU therapy, respectively. Data presented as median logHR (95% CI) of disease recurrence and progression after 3 years of follow-up. Abbreviations: HR: hazard ratio, CI: confidence interval.

Estimating the hazard of disease recurrence and progression for the levels of five miRNAs at baseline and after 3 and 6 months of treatment with 5-FU-based therapy (Fig. 5). A trend of increasing the hazard ratio toward the positive side was observed with miRNA19a-3p and miRNA17-5p, in which LogHR of − 0.14 and 0.13 were estimated for miRNA19a-3p and miRNA17-5p at baseline increased to be 0.01 and 0.28 after 3 months of treatment, respectively. Then at the end of therapy, the logHR increased to 0.66 and 0.5 for same both miRNA19a-3p and miRNA17-5p, respectively. That level of miRNA19a-3p at the end of treatment (6 months) was significantly associated with an increased hazard of disease recurrence and progression (*P* = 0.031).

## Discussion

CRC progress in a multistep pattern from early adenoma to late-stage adenocarcinoma through the accumulation of many genetic and epigenetic events initiated by APC mutation and chronic inflammation^21^. MiRNAs take place in this pathogenesis pathway either as promoters or inhibitors at all levels of CRC progression and at different expression levels^4,22–24^. Our 77 CRC patients showed significant upregulation of their serum miRNAs compared to matched sex and age-healthy controls. This high baseline expression of miRNAs had diagnostic potential and was associated with better median survival values. Web literature databases are rich with articles identifying the diagnostic and prognostic potentials of miRNAs in cancer in general and in CRC in particular^7,8,14,25–28^. Epigenetic markers like miRNAs retain the advantage of being changeable with treatment, and their levels in the peripheral blood of CRC patients almost match the corresponding levels in the tumors^29^. So, monitoring the pattern of change in their level during the treatment period will produce an honest reflection of disease progression and patient response to therapy^30,31^.

The use of 5-FU-based therapy is still the cornerstone in CRC treatment, despite the significant induction of its target, thymidylate synthase (TYMS), after 6 months of its usage, as we recorded before in a previous publication^32^. Digital karyotyping identified amplification of an approximately 100-kb region on chromosome 18p11.32 that contains the coding of TYMS in metastatic CRC patients subjected to prior treatment with 5-FU-based therapy^33^. This TYMS amplification is suggested to be controlled with some non-coding RNAs. Recently a review of long non-coding RNA (MALAT-1) highlighted its cooperation with co-factor complex (YAP1/TCF4/ β-catenin) to control specific groups of miRNAs and TYMS expression^34^.

Measuring the level of miRNAs with 5-FU-based therapy in this study exhibited a pattern of fluctuated expression from an initial reduction of their levels followed by an increase to the baseline or higher than the baseline level. It is known that 5-FU impacts RNA^35^. It causes splicing defects in intron-containing mRNA, rRNA, and tRNA leading to distorted transcription and post-transcriptional modification of uracil residues in RNA^35^. Tumor suppressor miRNAs, like miR375-3p, increased the chemosensitivity to 5-FU therapy through its direct targeting of TYMS^36^. Another suppressor miRNA, miRNA149, increased the chemosensitivity to 5-FU therapy by targeting Forkhead Box Transcription Factor (FOXM1)^37^. However, the oncogenic miRNA135b and miR-NA182 induced resistance to 5-FU by targeting ST6GALNAC2 via PI3K/AKT pathway^38^. So, the fluctuating changes of miRNAs with 5-FU therapy could be advantageous to monitor the patient's response to therapy and stratify them according to their treatment outcome.

Our data showed higher expression of miRNA223-3p level in patients ≤ 47 years than older age patients, matching the general association of reduced miRNA levels with age in healthy individuals. A study on 5221 healthy adults found that most peripheral miRNA levels were down expressed in older individuals^39^. In a microarray analysis of the serum of non-small cell lung cancer patients, miRNA223 was found to be down-expressed compared to healthy individuals, and its high level suppresses the expression of EGFR protein^40^. Such described tumor suppressor effect of miRNA223-3p matches our observation for the 3 months level of miRNA223-3p in which metastatic patients were showed to have lower miRNA223-3p compared to non-metastatic patients.

From the miRNAs which showed constant protective indication in its negative levels of logHR at baseline and after 5-FU-based therapy was miRNA20a-5p (logHR = − 0.23, − 0.11, − 1.64, baseline, 3 months, 6 months of 5-FU-based therapy respectively). Also, when its level reduced after 3 months of 5-FU-based therapy, that was associated with metastasis. In research conducted by Dalmasso et al. in 2014, the upregulation of miRNA20a-5p was related to gut microbiota-induced senescence to colonic cancer cells through targeting SUMO Specific Peptidase 1 (SENP1) and inducing the SUMOylation of P53^41^. The rest of the research suggests the pro-tumorigenic effects of miRNA20a leading to the induction of epithelial-mesenchymal transition (EMT)^42^ and regulation of TRAIL-induced apoptosis^43^. The treatment with 5-FU caused the fluctuated level of miRNA20a-5p by initial reduction, then induction. In our study, we claim that the 3 months level of miRNAs could correspond to the patients` sensitive phase to 5-FU-based therapy. Then the induction of miRNA levels after 6 months of 5-FU-based therapy could correspond to the phase at which CRC patients showed resistance to 5-FU-based therapy. The knockdown of miRNA20a sensitized CRC cell lines to cisplatin therapy through activation of ROS/ASK1/JNK pathway^44^, recommending its service as a follow-up response marker by Xiao et al.^45^.

In contrast, miRNA19a-3p showed a change in the association of its level before and after 5-FU-based therapy with the hazard of disease recurrence and progression. At baseline, the recorded logHR was = − 0.14 (95% CI = − 0.10–0.28), then after 3 and 6 months of therapy logHR increased to 0.01 and 0.66, respectively. That 6 months level showed significance with the increase in the hazard of disease recurrence and progression, *P* = 0.031. Interestingly, the reduced level of miRNA19a-3p after three months of therapy, which resemble the responsive phase of treatment, was associated with metastatic patients and those diagnosed at stage IV of the diagnosis, suggesting the initial tumor suppressor effect of that miRNA. However, the pattern of change of miRNA19a-3p level with therapy; of 3 months` reduction then 6 months` induction, showed significance in male patients, those with colonic site disease, and those with high ERK, EGFR, and PTEN protein levels, suggesting the conversion of miRNA19a-3p's function to be oncogenic during the resistant phase of treatment.

In a global miRNAs screen (miRNome) conducted by Uhlmann et al., they validate miRNA193a-3p along with miRNA124 and miRNA147 as tumor suppressors that co-target EGFR-driven breast cancer^46^. Similar co-operativity was seen between miRNA193a-3p and miRNA193a-5p to target EGFR in non-small cell lung cancer^47^, and the overexpression of miRNA19a exhibited anti-angiogenesis effects in inverse relation with KRAS expression in CRC cells^48^. The metastasis derived by miRNA19 in CRC is believed to happen because of the inhibition of transglutaminase-2^49^ and T-cell intracellular antigen 1^50^, suggesting an oncomeres` functions related to this miRNA. Another mRNA interaction site, testis-specific protein Y-encoded-like 5 (TSPYL5), which is related to miRNA19a as a target of suppression, leading to an increase in the aggressiveness of CRC disease^51^. The suppression of either miRNA19a or TSPYL5 induced cellular apoptosis and accumulation of HT29 cells at the G0/G1 phase of the cell cycle^51^. Finally, miRNA19a was found to be part of the construct of circulating exosomes in CRC patients^52^. Its high expression was associated with disease recurrence and overall poorer prognosis of the disease^52^.

Even when the hazard ratio was positive with the miRNA17a-5p level before and after therapy, its low level after 3 months of therapy was associated with metastasis like other miRNAs, which suggests the prevalence of the tumor suppressor function of miRNAs^53^. Most research measured miRNA17 suggesting its oncogenicity. Upregulation of miRNA17 was an indicator of poor prognosis in rectal cancer patients^54^, and in colon cancer cells through targeting Par4^55^, vimentin^56^, and CCNG2 in recurrent head and neck squamous cell carcinoma^57^.

CRC patients with low basal expression of PTEN and EGFR showed significantly higher expression of miRNA7-5p, which exhibited a fluctuated hazard ratio with 5-FU-based therapy of positive, then negative, and then to positive again. In concordance, the tumor suppressor function of miRNA7-5p was predominant in the literature. It has a direct effect on EGFR to suppress its level and an indirect effect on phospho-Akt to decrease tumor growth in vitro and in vivo^58^. Also, it was found that XRCC2, a key component in the homologous recombination repair pathway, is a target of miRNA7-5p, assisting in the apoptosis and chemo-sensitization of CRC cells^59^. So, the use of mimetics of miRNA7 was recommended to reduce multidrug resistance in various tumors^60^.

Patients with low ERK protein level (≤ 0.92), showed to have higher miRNA17a-5p and miRNA7-5p levels after 3 months of 5-FU-based therapy than the subgroup of patients with low baseline level of ERK. It is demonstrated that ERK has upstream effect on miRNAs through inhibiting pre-miRNA nuclear export^61^. Also, cap-binding protein 4EHP is found to be a mutual control of ERK phosphorylation and miRNA145 expression^62^. MiRNAs inhibition to endocytosis repressed embryonic stem cell differentiation through inhibition of ERK signaling^63^.

In general, 5-FU therapy-induced changes to the levels of circulating miRNAs associated with a certain subgroup of patients and with disease recurrence and progression. This pattern of change could be used as a marker of patients` response to therapy in studies recruiting larger number of patients. Also, further mechanistic studies should be conducted to explore this fluctuation of the tumor suppressor function of miRNAs over the treatment period. Is it a loss of function under the effect of 5-FU and/or oxaliplatin that could be reversed to sensitize CRC patients to treatment again or not?

## Conclusions

Serum miRNAs are measured in elevated levels in CRC patients before treatment with 5-FU-based therapy. From them, miRNA223-3p showed the best sensitivity, specificity, and AUC *diagnostic* parameters. Like most global epigenetic events such as DNA methylation^64^, 5-FU-based therapy changes the level of miRNAs over time. A significant trend of initial reduction and then increase was observed in miRNA223-3p, miRNA19a-3p, and miRNA7-5p after the 3 and 6 months of treatment with 5-FU-based therapy, respectively. That trend was significant with miRNA20a-5p and miRNA19a-3p in responder patients, and the change in miRNA19a-3p level over therapy was significant in patients with high basal EGFR, ERK, and PTEN protein levels. MiRNA223a-3p level was the most affected by adding oxaliplatin to the 5-FU treatment regimen.

At the 3 months sampling point, we found significant elevations in miRNA223-3p, miRNA20a-5p, miRNA19a-3p, and miRNA17-5p in non-metastatic patients relative to those diagnosed with metastasis. At the same time of 3 months, we found significant associations between high miRNA17-5p and miRNA7-5p with low ERK protein, and high miRNA17-5p level with low EGFR but high PTEN levels. This time point could be defined as the responsive phase to 5-FU-based therapy before experiencing the acute induction of TYMS as we showed before, and also suggest for the dominant tumor suppressor nature of miRNAs in cancer.

By moving to the 6 months treatment with 5-FU-based therapy, we noticed an increase in the hazard ratio of miRNA19a-3p, and its 6 months levels reached significance for predicting the hazard of disease recurrence and progression.

### Supplementary Information

Supplementary Information.

## Data Availability

The raw data analysed during the current study available from the corresponding author on reasonable request.
