# Supplementary material for: Rebound increase in microRNA levels at the end of 5-FU-based therapy in colorectal cancer patients
Source: Sci Rep. 2023 Aug 30;13:14237. doi: 10.1038/s41598-023-41030-7 (PMC10469181; doi:10.1038/s41598-023-41030-7)
Supplement: Supplementary file 1 — Supplementary Information. [file 41598_2023_41030_MOESM1_ESM.docx]

**Title:** Rebound increase in microRNA levels at the end of 5-FU-based therapy in colorectal cancer patients

**Table S1.** Clinicopathological features for the patients enrolled in the study at baseline and after 3 and 6 months of 5-FU-based therapy:

|  | | **Baseline** | **Three months of**  **5-FU therapy** | **Six months of**  **5-FU therapy** | **P- value** |
| --- | --- | --- | --- | --- | --- |
|  | | **Count (%)** | **Count (%)** | **Count (%)** |  |
|  | Total number of patients | 77 (100) | 60 (100) | 41 (100) |  |
| Age | >47 years | 40 (51.9) | 33 (55.0) | 23 (56.1) | 0.892 |
|  | <=47 years | 37 (48.1) | 27 (45.0) | 18 (43.9) |  |
| Sex | Female | 37 (48.1) | 25 (41.7) | 23 (56.1) | 0.361 |
|  | Male | 40 (51.9) | 35 (58.3) | 18 (43.9) |  |
| Diabetes mellitus | Undetermined | 2 (2.6) | 2 (3.3) | 1 (2.4) | 0.952 |
|  | Negative | 69 (89.6) | 55 (91.7) | 38 (92.7) |  |
|  | Positive | 6 (7.8) | 3 (5.0) | 2 (4.9) |  |
| Hypertension | Undetermined | 2 (2.6) | 2 (3.3) | 1 (2.4) | 0.994 |
|  | Negative | 67 (87.0) | 52 (86.7) | 35 (85.4) |  |
|  | Positive | 8 (10.4) | 6 (10.0) | 5 (12.2) |  |
| HCV infection | Undetermined | 2 (2.6) | 2 (3.3) | 1 (2.4) | 0.996 |
|  | Nega0tive | 69 (89.6) | 54 (90.0) | 36 (87.8) |  |
|  | Positive | 6 (7.8) | 4 (6.7) | 4 (9.8) |  |
| Smoking | Non-smoker | 55 (71.4) | 39 (65.0) | 30 (73.2) | 0.615 |
|  | Smoker | 22 (28.6) | 21 (35.0) | 11 (26.8) |  |
| Family history | Negative | 64 (83.1) | 51 (85.0) | 34 (82.9) | 0.945 |
|  | Positive | 13 (16.9) | 9 (15.0) | 7 (17.1) |  |
| Performance status | Undetermined | 2 (2.6) | 1 (1.7) | 1 (2.4) | 0.610 |
|  | 1 | 64 (83.1) | 53 (88.3) | 38 (92.7) |  |
|  | 2 | 11 (14.3) | 6 (10.0) | 2 (4.9) |  |
| CEA | Undetermined | 20 (26.0) | 16 (26.7) | 10 (24.4) | 0.593 |
|  | High | 22 (28.6) | 17 (28.3) | 7 (17.1) |  |
|  | Normal | 35 (45.5) | 27 (45.0) | 24 (58.5) |  |
| CA19.9 | Undetermined | 26 (33.8) | 20 (33.3) | 12 (29.3) | 0.559 |
|  | High | 10 (13.0) | 8 (13.3) | 2 (4.9) |  |
|  | Normal | 41 (53.2) | 32 (53.3) | 27 (65.9) |  |
| Treatment | Neoadjuvant | 49 (63.6) | 38 (63.4) | 26 (63.4) | 0.902 |
|  | Adjuvant | 7 (9.1) | 7 (11.6) | 6 (14.2) |  |
|  | Metastatic | 21 (27.3) | 15 (25.0) | 9 (21.4) |  |
| 5-FU therapy | Single | 19 (24.6) | 19 (31.6) | 18 (43.9) | 0.100 |
|  | Combination | 58 (75.3) | 41 (68.3) | 23 (70.0) |  |
| Site of tumor | Colon | 45 (58.4) | 37 (61.7) | 23 (56.1) | 0.848 |
|  | Rectum | 32 (41.6) | 23 (38.3) | 18 (43.9) |  |
| Pathology | Adenocarcinoma | 49 (63.6) | 40 (66.7) | 28 (68.3) | 0.978 |
|  | Adenocarcinoma, Mucinous | 16 (20.8) | 10 (16.7) | 8 (19.5) |  |
|  | Adenocarcinoma, Neuroendocrine | 1 (1.3) | 1 (1.7) | 1 (2.4) |  |
|  | Adenocarcinoma, Signet ring | 11 (14.3) | 9 (15.0) | 4 (9.8) |  |
| Grade | 2 | 59 (76.6) | 46 (76.7) | 34 (82.9) | 0.695 |
|  | 3 | 18 (23.4) | 14 (23.3) | 7 (17.1) |  |
| T | undetermined | 22 (28.6) | 15 (25.0) | 6 (14.6) | 0.665 |
|  | T2 | 12 (15.6) | 11 (18.3) | 10 (24.4) |  |
|  | T3 | 33 (42.9) | 28 (46.7) | 21 (51.2) |  |
|  | T4 | 10 (13.0) | 6 (10.0) | 4 (9.8) |  |
| N | Negative | 50 (64.9) | 40 (66.7) | 27 (65.9) | 0.977 |
|  | Positive | 27 (35.1) | 20 (33.3) | 14 (34.1) |  |
| M | Negative | 56 (72.7) | 45 (75.0) | 33 (80.5) | 0.647 |
|  | Positive | 21 (27.3) | 15 (25.0) | 8 (19.5) |  |
| Stage | II | 32 (41.6) | 27 (45.0) | 19 (46.3) | 0.941 |
|  | III | 21 (27.3) | 15 (25.0) | 12 (29.3) |  |
|  | IV | 24 (31.2) | 18 (30.0) | 10 (24.4) |  |
| Response to treatment | CR and SD | 42 (54.6) | 32 (53.3) | 21 (51.2) | 0.999 |
|  | PD | 24 (31.2) | 18 (30.0) | 13 (31.7) |  |
|  | Local recurrence | 5 (6.4) | 4 (6.6) | 3 (7.3) |  |
|  | Distance recurrence | 6 (7.8) | 6 (10.0) | 4 (9.7) |  |

Data presented as count and percentage of 77 CRC patients at different clinicopathological subgroups. **Abbreviations:** CEA: carcinoembryonic antigen; CA19.9: carbohydrate antigen 19.9; T: tumor burden; N: lymph node; M: metastasis, CR: complete response, SD: stable disease, PD: progressed disease.

**Table S2.** Effect of treatment regimen on the levels of miRNAs.

|  | **Baseline** | **3 months** | **6 months** | **P-value** |  |
| --- | --- | --- | --- | --- | --- |
|  | **miRNA223-3p** | | |  |  |
| Single (5-FU) | 346.59 (248.73-585.72) | 242.19 (153.61-455.09) | 593.15 (416.42- 1179.82) | 0.025 |  |
|  |  |  |  |  |  |
| Combination (+oxaliplatin) | 360.54 (124.02-872.88) | 132.01 (56.49-284.05) | 335.32 (161.58-674.29) | ***0.003*** |  |
|  |  |  |  |  |  |
| P-value | 0.787 | 0.045 | 0.032 |  |  |
|  | **miRNA20a-5p** | | |  |  |
| Single (5-FU) | 91.97 (72-320.04) | 83.29 (39.67-153.28) | 158.19 (97.14-302.29) | 0.204 |  |
|  |  |  |  |  |  |
| Combination (+oxaliplatin) | 68.13 (30.91-219.79) | 68.12 (23.1-112.99) | 101.83 (57.38-158.91) | 0.289 |  |
|  |  |  |  |  |  |
| P-value | 0.191 | 0.19 | 0.141 |  |  |
|  | **miRNA19a-3p** | | |  |  |
| Single (5-FU) | 34.15 (15.14- 89.89) | 16.68 (8.51-41.34) | 74.99 (33.9-121.73) | 0.08 |  |
|  |  |  |  |  |  |
| Combination (+oxaliplatin) | 26.32 (7.31-74.77) | 7.82 (4.59-28.84) | 36.3 (20.15-63.51) | 0.012 |  |
|  |  |  |  |  |  |
| P-value | 0.127 | 0.136 | 0.093 |  |  |
|  | **miRNA17-5p** | | |  |  |
| Single (5-FU) | 32.29 (22.46-105.78) | 30.27 (15.14-57.22) | 50.64 (38.35- 100.57) | 0.291 |  |
|  |  |  |  |  |  |
| Combination (+oxaliplatin) | 28.98 (10.16-83.7) | 22.01 (8.66-36.41) | 31.9 (19.42-58.05) | 0.289 |  |
|  |  |  |  |  |  |
| P-value | 0.339 | 0.13 | 0.06 |  |  |
|  | **miRNA7-5p** | | |  |  |
| Single (5-FU) | 2.51 (1.55-4.08) | 1.84 (0.79-3.26) | 3.69 (2.32-5.13) | 0.494 |  |
|  |  |  |  |  |  |
| Combination (+oxaliplatin) | 1.09 (0.57-5.05) | 1.25 (0.64-2.56) | 2.56 (1.56-4.38) | 0.054 |  |
|  |  |  |  |  |  |
| P-value | 0.159 | 0.322 | 0.17 |  |  |

Data are presented as median (IQR). Significant P-values ≤ 0.01 are displayed in bold- italic font. **Abbreviations:** 5-FU: 5-Fluorouracil.

**Table S3.** Level of miRNA223-3p in subgroups of CRC patients at baseline and after 3 and 6 months of 5-FU therapy:

| miRNA223-3p | | | | | | | | | | | | | | | | | |
| --- | --- | --- | --- | --- | --- | --- | --- | --- | --- | --- | --- | --- | --- | --- | --- | --- | --- |
| Subgroups | | | Baseline | | | | 3 months of 5-FU therapy | | | | | 6 months of 5-FU therapy | | | | | P-value |
|  |  |  | Median | | IQR | | Median | | IQR | | | Median | | IQR | | |  |
| Age | ≤47 years | 6.27 | | 5.58 | | 6.88 | 5.12 | 4.33 | | 5.65 | 6.03 | | 5.32 | | 6.91 | 0.037 | |
|  | >47 years | 5.68 | | 4.77 | | 6.17 | 5.38 | 4.24 | | 6.12 | 6.06 | | 5.52 | | 6.51 | 0.017 | |
| **P-value** | | | ***0.002*** | | | | 0.795 | | | | | 0.959 | | | | |  |
| Sex | Female | 5.98 | | 5.07 | | 6.54 | 5.26 | 4.67 | | 6.00 | 5.82 | | 5.24 | | 6.32 | 0.260 | |
|  | Male | 5.81 | | 5.36 | | 6.67 | 5.20 | 4.03 | | 6.07 | 6.58 | | 5.92 | | 7.08 | ***<0.001*** | |
| **P-value** | | | 0.959 | | | | 0.584 | | | | | 0.013 | | | | |  |
| Smoking | Non-smoker | 5.89 | | 5.24 | | 6.54 | 5.20 | 4.60 | | 6.00 | 5.90 | | 5.34 | | 6.39 | 0.079 | |
|  | Smoker | 5.83 | | 5.02 | | 6.93 | 5.28 | 4.14 | | 6.09 | 6.80 | | 6.16 | | 7.15 | ***0.004*** | |
| **P-value** | | | 0.901 | | | | 0.957 | | | | | 0.012 | | | | |  |
| Site of tumor | colon | 5.98 | | 5.50 | | 6.57 | 5.28 | 4.24 | | 5.82 | 6.15 | | 5.37 | | 6.76 | ***0.002*** | |
|  | Rectum | 5.72 | | 4.82 | | 6.54 | 5.20 | 4.60 | | 6.12 | 6.03 | | 5.54 | | 6.89 | 0.348 | |
| **P-value** | | | 0.301 | | | | 0.721 | | | | | 0.799 | | | | |  |
| T | T2 | 6.12 | | 5.31 | | 6.56 | 5.20 | 4.63 | | 6.41 | 6.35 | | 5.94 | | 6.51 | 0.150 | |
|  | T3 | 6.07 | | 5.52 | | 6.78 | 5.24 | 3.91 | | 5.95 | 5.96 | | 5.04 | | 6.71 | ***<0.001*** | |
|  | T4 | 5.90 | | 5.29 | | 6.77 | 5.33 | 5.26 | | 5.50 | 6.53 | | 5.56 | | 7.41 | 0.472 | |
| **P-value** | | | 0.197 | | | | 0.578 | | | | | 0.632 | | | | |  |
| N | Negative | 5.79 | | 4.82 | | 6.37 | 5.29 | 4.49 | | 6.16 | 6.08 | | 5.54 | | 6.82 | 0.067 | |
|  | Positive | 6.17 | | 5.50 | | 6.93 | 5.04 | 4.19 | | 5.57 | 5.82 | | 5.01 | | 7.03 | ***0.001*** | |
| **P-value** | | | 0.094 | | | | 0.301 | | | | | 0.276 | | | | |  |
| M | Negative | 5.96 | | 5.51 | | 6.65 | 5.48 | 4.67 | | 6.12 | 6.05 | | 5.52 | | 6.82 | 0.013 | |
|  | Positive | 5.41 | | 4.58 | | 6.34 | 4.33 | 3.66 | | 5.11 | 5.81 | | 5.21 | | 6.58 | 0.093 | |
| **P-value** | | | 0.036 | | | | ***0.004*** | | | | | 0.414 | | | | |  |
| Stage | II | 5.92 | | 5.64 | | 6.54 | 5.55 | 5.03 | | 6.31 | 6.39 | | 5.94 | | 6.82 | 0.104 | |
|  | III | 6.17 | | 5.50 | | 6.78 | 4.98 | 4.14 | | 5.65 | 5.71 | | 5.01 | | 6.26 | 0.012 | |
|  | IV | 5.25 | | 4.53 | | 6.27 | 4.35 | 3.68 | | 5.30 | 5.85 | | 5.34 | | 6.89 | 0.150 | |
| **P-value** | | | 0.040 | | | | ***0.007*** | | | | | 0.228 | | | | |  |

Data presented as medians and IQR of miRNA223-3p level. Significant P-values ≤ 0.01 are displayed in bold- italic font. **Abbreviations:** IQR: interquartile range, T: tumor burden, N: lymph node, M: metastasis.

**Table S4.** Level of miRNA20a-5p in subgroups of CRC patients at baseline and after 3 and 6 months of 5-FU therapy:

| miRNA20a-5p | | | | | | | | | | | |
| --- | --- | --- | --- | --- | --- | --- | --- | --- | --- | --- | --- |
| Subgroups | | Baseline | | | 3 months of 5-FU therapy | | | 6 months of 5-FU therapy | | | **P-value** |
|  |  | Median | IQR | | Median | IQR | | Median | IQR | |  |
| Age | ≤47 years | 4.70 | 3.90 | 5.81 | 4.22 | 3.47 | 4.50 | 4.83 | 4.39 | 5.87 | 0.048 |
|  | >47 years | 4.34 | 3.43 | 5.18 | 4.55 | 3.32 | 5.03 | 4.84 | 4.05 | 5.31 | 0.846 |
| **P-value** | | 0.073 | | | 0.422 | | | 0.559 | | |  |
| Sex | Female | 4.66 | 3.66 | 5.85 | 4.29 | 3.60 | 4.74 | 4.62 | 4.05 | 5.10 | 0.593 |
|  | Male | 4.45 | 3.69 | 5.43 | 4.30 | 3.14 | 5.03 | 5.05 | 4.52 | 6.02 | 0.076 |
| **P-value** | | 0.632 | | | 0.759 | | | 0.114 | | |  |
| Smoking | Non-smoker | 4.52 | 3.66 | 5.58 | 4.29 | 3.53 | 4.86 | 4.62 | 4.13 | 5.10 | 0.393 |
|  | Smoker | 4.45 | 3.59 | 5.47 | 4.30 | 3.22 | 5.03 | 5.18 | 4.95 | 6.11 | 0.125 |
| **P-value** | | 0.660 | | | 0.859 | | | 0.077 | | |  |
| Site of tumor | colon | 4.52 | 3.75 | 5.22 | 4.45 | 3.14 | 5.00 | 4.94 | 4.41 | 5.79 | 0.214 |
|  | Rectum | 4.40 | 3.45 | 5.67 | 4.22 | 3.57 | 4.55 | 4.69 | 4.13 | 5.10 | 0.311 |
| **P-value** | | 0.502 | | | 0.927 | | | 0.559 | | |  |
| T | T2 | 4.66 | 4.43 | 6.27 | 4.42 | 3.47 | 5.03 | 4.59 | 4.52 | 5.43 | 0.122 |
|  | T3 | 4.78 | 4.19 | 5.58 | 4.31 | 3.50 | 4.94 | 4.78 | 4.03 | 5.71 | 0.422 |
|  | T4 | 3.98 | 3.30 | 4.87 | 4.36 | 3.52 | 5.00 | 5.27 | 4.64 | 5.94 | 0.174 |
| **P-value** | | 0.048 | | | 0.466 | | | 0.528 | | |  |
| N | Negative | 4.40 | 3.32 | 5.58 | 4.42 | 3.47 | 5.01 | 4.93 | 4.58 | 5.61 | 0.203 |
|  | Positive | 4.74 | 3.96 | 5.47 | 3.86 | 3.40 | 4.58 | 4.62 | 3.92 | 5.86 | 0.420 |
| **P-value** | | 0.326 | | | 0.355 | | | 0.351 | | |  |
| M | Negative | 4.66 | 3.92 | 5.68 | 4.42 | 3.68 | 5.00 | 4.94 | 4.13 | 5.71 | 0.217 |
|  | Positive | 3.92 | 2.68 | 4.65 | 3.32 | 2.59 | 4.49 | 4.72 | 4.62 | 5.01 | 0.325 |
| **P-value** | | 0.017 | | | ***0.005*** | | | 0.432 | | |  |
| Stage | II | 4.66 | 3.92 | 6.06 | 4.45 | 3.68 | 5.03 | 4.95 | 4.56 | 5.71 | 0.143 |
|  | III | 4.78 | 4.08 | 5.47 | 4.29 | 3.42 | 5.13 | 4.27 | 3.87 | 5.05 | 0.926 |
|  | IV | 4.01 | 2.69 | 4.70 | 3.35 | 2.59 | 4.49 | 4.85 | 4.62 | 5.10 | 0.150 |
| **P-value** | | 0.033 | | | 0.015 | | | 0.241 | | |  |

Data presented as medians and IQR of miRNA20a-5p level. Significant P-values ≤ 0.01 are displayed in bold- italic font. **Abbreviations:** IQR: interquartile range, T: tumor burden, N: lymph node, M: metastasis.

**Table S5.** Level of miRNA19a-3p in subgroups of CRC patients at baseline and after 3 and 6 months of 5-FU therapy:

| miRNA19a-3p | | | | | | | | | | | |
| --- | --- | --- | --- | --- | --- | --- | --- | --- | --- | --- | --- |
| Subgroups | | Baseline | | | 3 months of 5-FU therapy | | | 6 months of 5-FU therapy | | | P- value |
|  |  | Median | IQR | | Median | IQR | | Median | IQR | |  |
| Age | <=47 years | 3.64 | 2.72 | 4.55 | 2.47 | 1.55 | 3.24 | 3.87 | 3.33 | 4.79 | 0.016 |
|  | >47 years | 2.84 | 2.04 | 4.12 | 2.80 | 1.98 | 3.72 | 3.75 | 3.24 | 4.62 | 0.066 |
| **P-value** | | 0.058 | | | 0.249 | | | 0.666 | | |  |
| Sex | Female | 3.53 | 2.39 | 4.34 | 2.51 | 1.75 | 3.42 | 3.53 | 3.00 | 4.62 | 0.154 |
|  | Male | 3.34 | 2.32 | 4.41 | 2.57 | 1.43 | 3.58 | 4.26 | 3.52 | 4.98 | ***0.003*** |
| **P-value** | | 0.791 | | | 0.725 | | | 0.093 | | |  |
| Smoking | Non-smoker | 3.24 | 2.27 | 4.35 | 2.51 | 1.72 | 3.38 | 3.56 | 3.24 | 4.60 | 0.016 |
|  | Smoker | 3.53 | 2.51 | 4.50 | 2.58 | 1.25 | 3.70 | 4.38 | 4.04 | 5.00 | 0.039 |
| **P-value** | | 0.883 | | | 0.883 | | | 0.104 | | |  |
| Site of tumor | colon | 3.59 | 2.36 | 4.35 | 2.47 | 1.57 | 3.70 | 3.89 | 3.31 | 4.63 | ***0.004*** |
|  | Rectum | 2.94 | 2.29 | 4.42 | 2.58 | 1.75 | 3.24 | 3.67 | 3.00 | 4.56 | 0.179 |
| **P-value** | | 0.790 | | | 0.684 | | | 0.552 | | |  |
| T | T2 | 3.54 | 2.88 | 4.80 | 2.81 | 1.57 | 4.13 | 3.41 | 3.10 | 4.60 | 0.273 |
|  | T3 | 3.77 | 2.83 | 4.45 | 2.54 | 1.58 | 3.64 | 3.75 | 3.24 | 4.45 | 0.015 |
|  | T4 | 2.70 | 1.99 | 3.86 | 2.92 | 1.98 | 3.38 | 4.35 | 3.28 | 5.31 | 0.472 |
| **P-value** | | 0.028 | | | 0.369 | | | 0.457 | | |  |
| N | Negative | 2.95 | 2.05 | 4.34 | 2.81 | 1.53 | 3.71 | 3.86 | 3.29 | 4.63 | 0.045 |
|  | Positive | 3.63 | 2.46 | 4.43 | 2.32 | 1.78 | 3.09 | 3.63 | 3.00 | 4.45 | 0.011 |
| **P-value** | | 0.286 | | | 0.393 | | |  | 0.454 |  |  |
| M | Negative | 3.61 | 2.66 | 4.46 | 2.81 | 2.06 | 3.70 | 3.89 | 3.24 | 4.63 | 0.014 |
|  | Positive | 2.19 | 1.70 | 3.64 | 1.72 | 0.78 | 1.98 | 3.73 | 3.56 | 4.59 | 0.044 |
| **P-value** | | 0.022 | | | ***0.002*** | | | 0.468 | | |  |
| Stage | II | 3.66 | 2.77 | 4.86 | 3.03 | 2.30 | 3.92 | 3.92 | 3.27 | 4.80 | 0.150 |
|  | III | 3.73 | 2.45 | 4.35 | 2.50 | 1.98 | 3.36 | 3.38 | 3.00 | 4.32 | 0.116 |
|  | IV | 2.35 | 1.68 | 3.59 | 1.73 | 0.81 | 2.78 | 3.87 | 3.59 | 4.62 | 0.020 |
| **P-value** | | 0.015 | | | ***0.005*** | | | 0.322 | | |  |

Data presented as medians and IQR of miRNA19a-3p level. Significant P-values ≤ 0.01 are displayed in bold- italic font. **Abbreviations:** IQR: interquartile range, T: tumor burden, N: lymph node, M: metastasis.

**Table S6.** Level of miRNA17-5p in subgroups of CRC patients at baseline and after 3 and 6 months of 5-FU therapy:

| miRNA17-5p | | | | | | | | | | | |
| --- | --- | --- | --- | --- | --- | --- | --- | --- | --- | --- | --- |
| Subgroups | | Baseline | | | 3 months of 5-FU therapy | | | 6 months of 5-FU therapy | | | P-value |
|  |  | Median | IQR | | Median | IQR | | Median | IQR | |  |
| Age | <=47 years | 3.63 | 2.95 | 4.89 | 3.04 | 2.49 | 3.59 | 3.68 | 3.28 | 4.86 | 0.093 |
|  | >47 years | 3.29 | 2.32 | 3.89 | 3.41 | 2.26 | 4.05 | 3.71 | 2.97 | 4.14 | 0.678 |
| **P-value** | | 0.038 | | | 0.431 | | | 0.647 | | |  |
| Sex | Female | 3.61 | 2.56 | 4.89 | 3.16 | 2.72 | 3.59 | 3.49 | 2.97 | 4.11 | 0.499 |
|  | Male | 3.39 | 2.60 | 4.32 | 3.16 | 2.16 | 4.05 | 3.92 | 3.46 | 4.89 | 0.150 |
| **P-value** | | 0.823 | | | 0.708 | | | 0.103 | | |  |
| Smoking | Non-smoker | 3.47 | 2.56 | 4.76 | 3.16 | 2.52 | 3.76 | 3.50 | 3.09 | 4.11 | 0.435 |
|  | Smoker | 3.39 | 2.57 | 4.29 | 3.16 | 2.16 | 3.94 | 4.10 | 3.82 | 4.98 | 0.125 |
| **P-value** | | 0.725 | | | 0.926 | | | 0.064 | | |  |
| Site of tumor | colon | 3.47 | 2.83 | 4.15 | 3.41 | 2.16 | 3.94 | 3.78 | 3.31 | 4.72 | 0.275 |
|  | Rectum | 3.29 | 2.42 | 4.70 | 3.04 | 2.56 | 3.41 | 3.51 | 3.09 | 4.11 | 0.311 |
| **P-value** | | 0.476 | | |  | 0.761 |  | 0.542 | | |  |
| T | T2 | 3.66 | 3.20 | 5.12 | 3.31 | 2.72 | 3.94 | 3.58 | 3.46 | 4.38 | 0.301 |
|  | T3 | 3.64 | 3.17 | 4.66 | 3.39 | 2.61 | 3.96 | 3.62 | 2.90 | 4.61 | 0.422 |
|  | T4 | 2.95 | 2.20 | 3.74 | 3.32 | 2.47 | 4.05 | 4.23 | 3.48 | 4.89 | 0.174 |
| **P-value** | | 0.042 | | | 0.323 | | | 0.614 | | |  |
| N | Negative | 3.29 | 2.32 | 4.43 | 3.34 | 2.48 | 3.97 | 3.78 | 3.46 | 4.58 | 0.203 |
|  | Positive | 3.62 | 2.97 | 4.66 | 2.87 | 2.49 | 3.49 | 3.42 | 2.88 | 4.88 | 0.420 |
| **P-value** | | 0.224 | | | 0.363 | | | 0.351 | | |  |
| M | Negative | 3.62 | 2.94 | 4.75 | 3.37 | 2.72 | 3.94 | 3.78 | 3.09 | 4.61 | 0.237 |
|  | Positive | 2.67 | 1.80 | 3.61 | 2.47 | 2.05 | 3.49 | 3.54 | 3.47 | 3.94 | 0.325 |
| **P-value** | | 0.016 | | | ***0.007*** | | | 0.673 | | |  |
| Stage | II | 3.64 | 2.94 | 4.83 | 3.41 | 2.72 | 4.05 | 3.78 | 3.46 | 4.61 | 0.196 |
|  | III | 3.64 | 2.98 | 4.66 | 3.16 | 2.56 | 4.19 | 3.20 | 2.88 | 4.06 | 0.926 |
|  | IV | 2.88 | 1.75 | 3.61 | 2.49 | 2.05 | 3.49 | 3.75 | 3.48 | 4.11 | 0.150 |
| **P-value** | | 0.034 | | | 0.023 | | | 0.246 | | |  |

Data presented as medians and IQR of miRNA17-5p level. Significant P-values ≤ 0.01 are displayed in bold- italic font. **Abbreviations:** IQR: interquartile range, T: tumor burden, N: lymph node, M: metastasis.

**Table S7.** Level of miRNA7-5p in subgroups of CRC patients at baseline and after 3 and 6 months of 5-FU therapy:

| miRNA7-5p | | | | | | | | | | | |
| --- | --- | --- | --- | --- | --- | --- | --- | --- | --- | --- | --- |
| Subgroups | | Baseline | | | 3 months of 5-FU therapy | | | 6 months of 5-FU therapy | | | P-value |
|  |  | Median | IQR | | Median | IQR | | Median | IQR | |  |
| Age | ≤47 years | 0.65 | -0.28 | 1.73 | 0.13 | -0.48 | 1.02 | 1.07 | 0.59 | 1.60 | ***0.006*** |
|  | >47 years | 0.67 | -0.42 | 1.50 | 0.47 | -0.13 | 1.18 | 0.93 | 0.24 | 1.52 | 0.946 |
| **P-value** | | 0.548 | | | 0.275 | | | 0.416 | | |  |
| Sex | Female | 1.05 | -0.40 | 1.95 | 0.51 | -0.13 | 1.09 | 0.94 | 0.54 | 1.52 | 0.200 |
|  | Male | 0.54 | -0.28 | 1.04 | 0.18 | -0.59 | 1.06 | 1.06 | 0.48 | 1.64 | 0.128 |
| **P-value** | | 0.191 | | | 0.277 | | | 0.622 | | |  |
| Smoking | Non-smoker | 0.67 | -0.42 | 1.73 | 0.50 | -0.31 | 1.09 | 0.94 | 0.48 | 1.52 | 0.177 |
|  | Smoker | 0.65 | -0.22 | 1.01 | 0.13 | -0.59 | 1.01 | 1.08 | 0.88 | 1.80 | 0.174 |
| **P-value** | | 0.468 | | | 0.299 | | | 0.500 | | |  |
| Site of tumor | colon | 0.62 | -0.33 | 1.37 | 0.43 | -0.31 | 1.06 | 0.88 | 0.47 | 1.56 | 0.167 |
|  | Rectum | 0.93 | -0.42 | 1.73 | 0.13 | -0.45 | 1.09 | 1.13 | 0.92 | 1.56 | 0.211 |
| **P-value** | | 0.552 | | | 0.891 | | | 0.334 | | |  |
| T | T2 | 1.33 | 0.53 | 2.35 | 0.66 | -0.23 | 1.06 | 0.88 | 0.54 | 1.56 | 0.202 |
|  | T3 | 0.92 | -0.17 | 1.62 | 0.30 | -0.39 | 1.10 | 1.06 | 0.44 | 1.56 | 0.195 |
|  | T4 | 0.01 | -0.37 | 0.82 | 0.54 | -0.01 | 1.13 | 0.95 | 0.72 | 1.78 | 0.039 |
| **P-value** | | 0.041 | | | 0.792 | | | 0.642 | | |  |
| N | Negative | 0.64 | -0.46 | 1.45 | 0.54 | -0.39 | 1.15 | 1.19 | 0.74 | 1.64 | 0.146 |
|  | Positive | 0.85 | -0.19 | 1.61 | 0.13 | -0.38 | 0.54 | 1.06 | 0.02 | 1.17 | 0.165 |
| **P-value** | | 0.661 | | | 0.276 | | | 0.131 | | |  |
| M | Negative | 0.82 | -0.04 | 1.62 | 0.47 | -0.23 | 1.13 | 1.06 | 0.48 | 1.56 | 0.157 |
|  | Positive | -0.15 | -0.77 | 0.94 | -0.01 | -0.71 | 0.58 | 1.12 | 0.87 | 1.50 | 0.093 |
| **P-value** | | 0.075 | | | 0.583 | | | 0.332 | | |  |
| Stage | II | 0.67 | 0.02 | 1.62 | 0.61 | -0.23 | 1.18 | 1.19 | 0.64 | 1.74 | 0.268 |
|  | III | 1.01 | -0.28 | 1.81 | 0.22 | -0.31 | 1.02 | 0.55 | 0.24 | 1.08 | 0.368 |
|  | IV | -0.12 | -0.75 | 1.10 | 0.04 | -0.71 | 0.84 | 1.24 | 0.94 | 1.52 | 0.150 |
| **P-value** | | 0.216 | | | 0.169 | | | 0.102 | | |  |

Data presented as medians and IQR of miRNA7-5p level. Significant P-values ≤ 0.01 are displayed in bold- italic font. **Abbreviations:** IQR: interquartile range, T: tumor burden, N: lymph node, M: metastasis.

**Table S8:** Change in the miRNAs level in subgroups of CRC patients at the end of treatment with 5-FU based therapy:

| Subgroups of patients | | Change in miRNA223-3p | | P-value | Change in miRNA20a-5p | | P-value | Change in miRNA19a-3p | | P-value |
| --- | --- | --- | --- | --- | --- | --- | --- | --- | --- | --- |
|  |  | Increase | Decrease |  | Increase | Decrease |  | Increase | Decrease |  |
|  |  | Count (%) | Count (%) |  | Count (%) | Count (%) |  | Count (%) | Count (%) |  |
| Age | ≤47 years | 16 (51.60) | 8 (72.70) | 0.294 | 14 (63.60) | 10 (50.00) | 0.411 | 14 (58.30) | 10 (55.60) | 0.597 |
|  | >47 years | 15 (48.40) | 3 (27.30) |  | 8 (36.40) | 10 (50.00) |  | 10 (41.70) | 8 (44.40) |  |
| Sex | Female | 13 (41.90) | 10 (90.90) | ***0.009*** | 10 (45.50) | 13 (65.00) | 0.195 | 13 (54.20) | 10 (55.60) | 0.433 |
|  | Male | 18 (85.10) | 1 (9.10) |  | 12 (54.50) | 7 (35.00) |  | 11 (45.80) | 8 (44.40) |  |
| Smoking | Non-smoker | 19 (61.30) | 11 (100) | 0.051 | 15 (68.20) | 15 (75.00) | 0.888 | 18 (75.00) | 12 (66.70) | 0.839 |
|  | Smoker | 12 (38.70) |  |  | 7 (31.80) | 5 (25.00) |  | 6 (25.00) | 6 (33.30) |  |
| CEA | High | 6 (19.40) | 2 (18.20) | 0.185 | 7 (31.80) | 1 (5.00) | 0.048 | 7 (29.00) | 1 (5.60) | 0.059 |
|  | Normal | 17 (54.80) | 7 (63.60) |  | 11 (50.00) | 13 (65.00) |  | 13 (54.20) | 11 (61.10) |  |
| CA19.9 | High | 2 (6.50) |  | 0.06 | 1 (4.50) | 1 (5.00) | 0.025 | 1 (4.20) | 1 (5.60) | 0.034 |
|  | Normal | 20 (64.50) | 8 (72.70) |  | 17 (77.30) | 11 (55.00) |  | 18 (75.00) | 10 (55.60) |  |
| Site of tumor | colon | 21 (67.70) | 3 (27.30) | 0.063 | 12 (54.50) | 12 (60.00) | 0.908 | 14 (58.30) | 10 (55.60) | 0.953 |
|  | Rectum | 10 (32.30) | 8 (72.70) |  | 10 (45.50) | 8 (40.00) |  | 10 (41.70) | 8 (44.40) |  |
| T | T2 | 7 (22.60) | 3 (27.30) | 0.032 | 3 (13.60) | 7 (35.00) | ***0.006*** | 5 (20.80) | 5(27.80) | 0.017 |
|  | T3 | 17 (54.80) | 5 (45.50) |  | 11 (50.00) | 11 (55.00) |  | 11 (45.80) | 11 (61.10) |  |
|  | T4 | 3 (9.70) | 1 (9.10) |  | 3 (13.60) | 1 (5.00) |  | 3 (12.50) | 1 (5.60) |  |
| LN | Negative | 19 (61.30) | 8 (72.70) | 0.785 | 14 (63.60) | 13 (65.00) | 0.987 | 16 (66.70) | 11 (61.10) | 0.925 |
|  | Positive | 12 (38.70) | 3 (27.30) |  | 8 (36.40) | 7 (35.00) |  | 8 (33.30) | 7 (38.90) |  |
| M | Negative | 24 (77.40) | 10 (90.90) | 0.143 | 17 (77.30) | 17 (85.00) | 0.177 | 19 (79.20) | 15 (83.30) | 0.198 |
|  | Positive | 7 (22.60) | 1 (9.10) |  | 5 (22.70) | 3 (15.00) |  | 5 (20.80) | 3 (16.70) |  |
| Stage | II | 13 (41.90) | 6 (54.50) | 0.574 | 9 (40.90) | 10 (50.00) | 0.440 | 11 (45.80) | 8 (44.40) | 0.461 |
|  | III | 10 (32.30) | 3 (27.30) |  | 6 (27.30) | 7 (35.00) |  | 6 (25.00) | 7 (38.90) |  |
|  | IV | 8 (25.80) | 2 (18.20) |  | 7 (31.80) | 3 (15.00) |  | 7 (29.20) | 3 (16.70) |  |

Data presented as count and % of patients in subgroups according to the change in their miRNAs levels after 6 months of 5-FU based therapy relative to baseline. Significant P-values ≤ 0.01 are displayed in bold- italic font. **Abbreviations:** CEA, carcinoembryonic antigen; CA19.9, carbohydrate antigen 19.9, T: tumor burden, N: lymph node, M: metastasis.

**Table S9:** Change in the miRNAs level in subgroups of CRC patients at the end of treatment with 5-FU based therapy:

| Subgroups of patients | | Change in miRNA17-5p | | P-value | Change in miRNA7-5p | | P-value |
| --- | --- | --- | --- | --- | --- | --- | --- |
|  |  | Increase | Decrease |  | Increase | Decrease |  |
|  |  | Count (%) | Count (%) |  | Count (%) | Count (%) |  |
| Age | ≤47 years | 13 (56.50) | 11 (57.90) | 0.604 | 15 (62.50) | 9 (50.00) | 0.440 |
|  | >47 years | 10 (43.50) | 8 (42.10) |  | 9 (37.50) | 9 (50.00) |  |
| Sex | Female | 12 (52.20) | 11 (57.90) | 0.406 | 10 (41.70) | 13 (72.20) | 0.063 |
|  | Male | 11 (47.80) | 8 (42.10) |  | 14 (58.30) | 5 (27.80) |  |
| Smoking | Non-smoker | 16 (69.60) | 14 (73.70) | 0.958 | 15 (62.50) | 15 (83.30) | 0.335 |
|  | Smoker | 7 (30.40) | 5 (26.30) |  | 9 (37.50) | 3 (16.70) |  |
| CEA | High | 7 (30.40) | 1 (5.30) | 0.055 | 6 (25.00) | 2 (11.10) | 0.124 |
|  | Normal | 12 (52.20) | 12 (63.20) |  | 12 (50.00) | 12 (66.70) |  |
| CA19.9 | High | 1 (4.30) | 1 (5.30) | 0.019 | 1 (4.20) | 1 (5.60) | 0.059 |
|  | Normal | 18 (78.30) | 10 (52.60) |  | 17 (70.80) | 11 (61.10) |  |
| Site of tumor | colon | 13 (56.50) | 11 (57.90) | 0.965 | 15 (62.50) | 9 (50.00) | 0.696 |
|  | Rectum | 10 (43.50) | 8 (42.10) |  | 9 (37.50) | 9 (50.00) |  |
| T | T2 | 4 (17.40) | 6 (31.60) | 0.011 | 3 (12.50) | 7 (38.90) | ***0.002*** |
|  | T3 | 11 (47.80) | 11 (57.90) |  | 13 (54.20) | 9 (50.00) |  |
|  | T4 | 3 (13.00) | 1 (5.30) |  | 4 (16.70) |  |  |
| LN | Negative | 15 (65.20) | 12 (63.20) | 0.982 | 16 (66.70) | 11 (61.10) | 0.925 |
|  | Positive | 8 (34.80) | 7 (36.80) |  | 8 (33.30) | 7 (38.90) |  |
| M | Negative | 18 (78.30) | 16 (84.20) | 0.189 | 17 (70.80) | 17 (94.40) | 0.049 |
|  | Positive | 5 (21.70) | 3 (15.80) |  | 7 (29.20) | 1 (5.60) |  |
| Stage | II | 10 (43.50) | 9 (47.40) | 0.469 | 10 (41.70) | 9 (50.00) | 0.298 |
|  | III | 6 (26.10) | 7 (36.80) |  | 6 (25.00) | 7 (38.90) |  |
|  | IV | 7 (30.40) | 3 (15.80) |  | 8 (33.30) | 2 (11.10) |  |

Data presented as count and % of patients in subgroups according to the change in their miRNAs levels after 6 months of 5-FU based therapy relative to baseline. Significant P-values ≤ 0.01 are displayed in bold- italic font. **Abbreviations:** CEA, carcinoembryonic antigen; CA19.9, carbohydrate antigen 19.9, T: tumor burden, N: lymph node, M: metastasis.


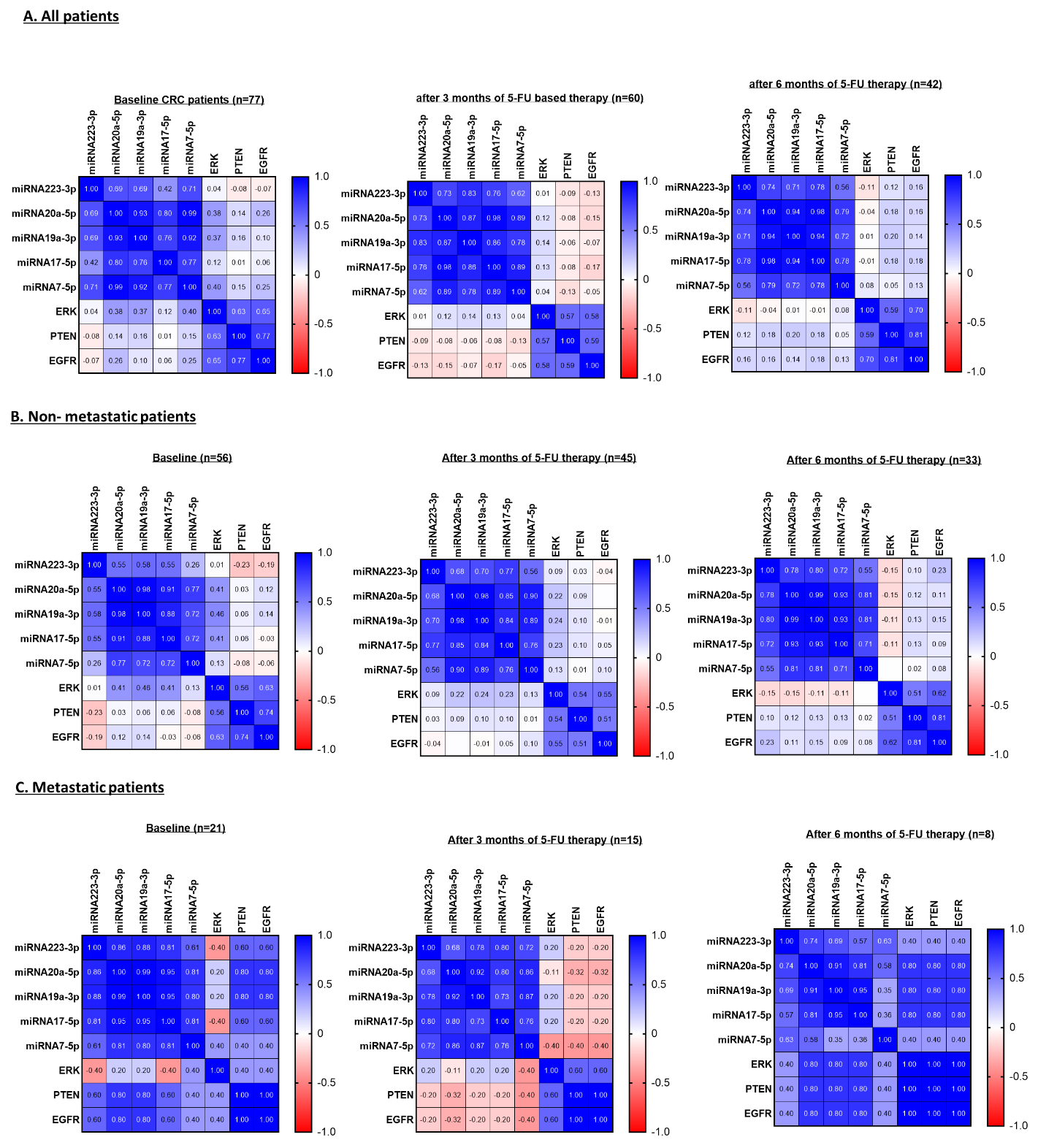


**Figure S1.** The correlation between the miRNA levels (miRNA223-3p, miRNA20a.5p, miRNA17-5p, miRNA19a-3P, miRNA7-5p) and proteins (ERK, PTEN, and EGFR) at baseline, and after 3 months and 6 months of 5FU therapy in **A.** all CRC patients, **B.** non-metastatic patients, and **C.** metastatic patients.


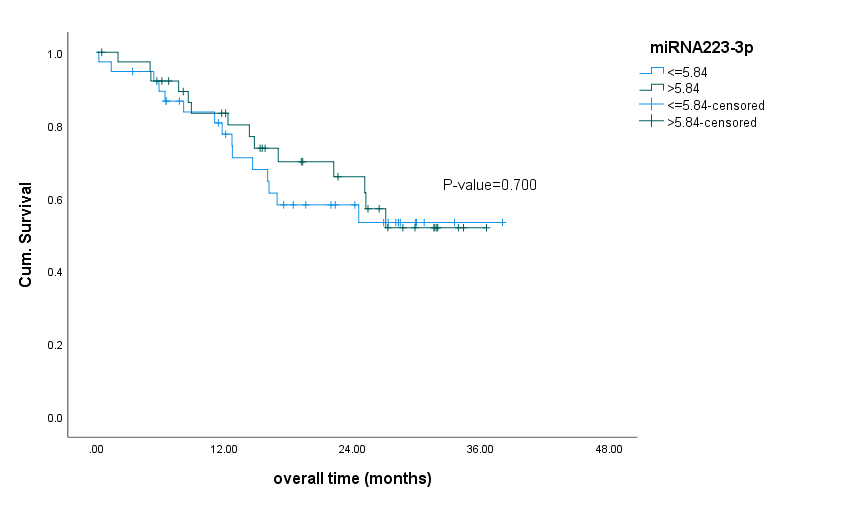


**Figure S2.** Effect of miRNA223-3p on the OS of 77 CRC patients before 5-FU therapy.


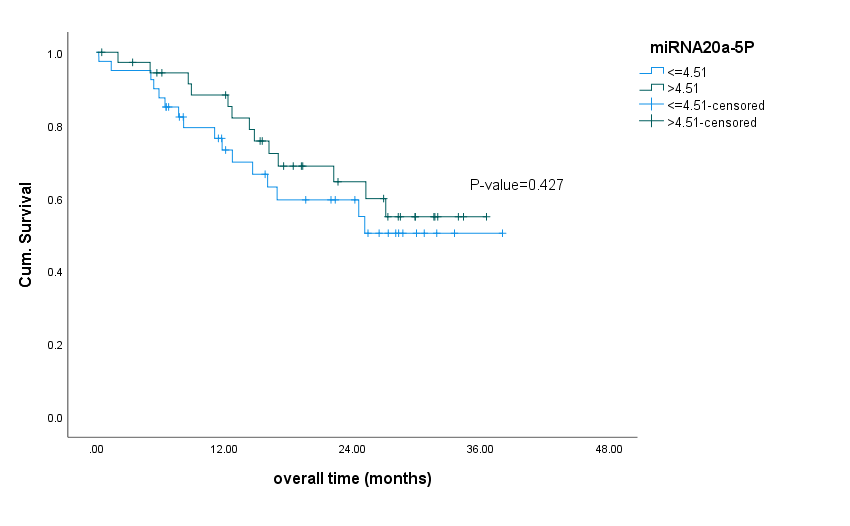


**Figure S3.** Effect of miRNA20a-5p on the OS of 77 CRC patients before 5-FU therapy.


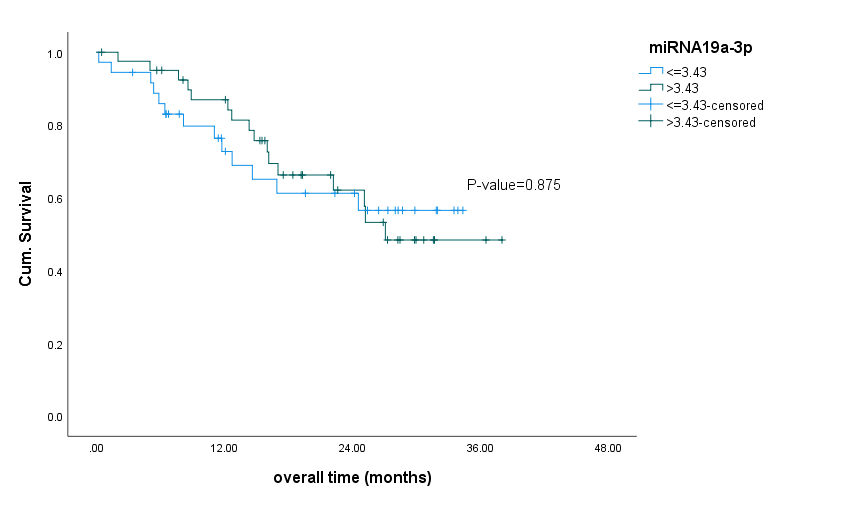


**Figure S4.** Effect of miRNA19a-3p on the OS of 77 CRC patients before 5-FU therapy.


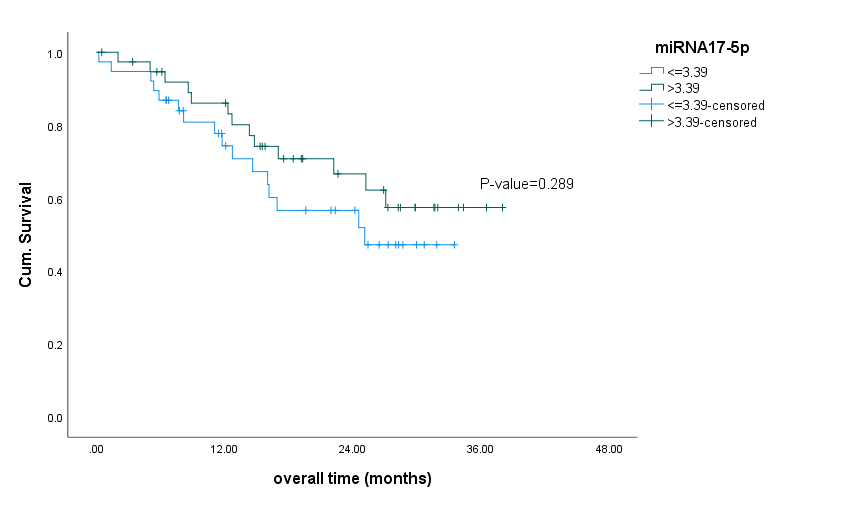


**Figure S5.** Effect of miRNA17-5p on the OS of 77 CRC patients before 5-FU therapy.


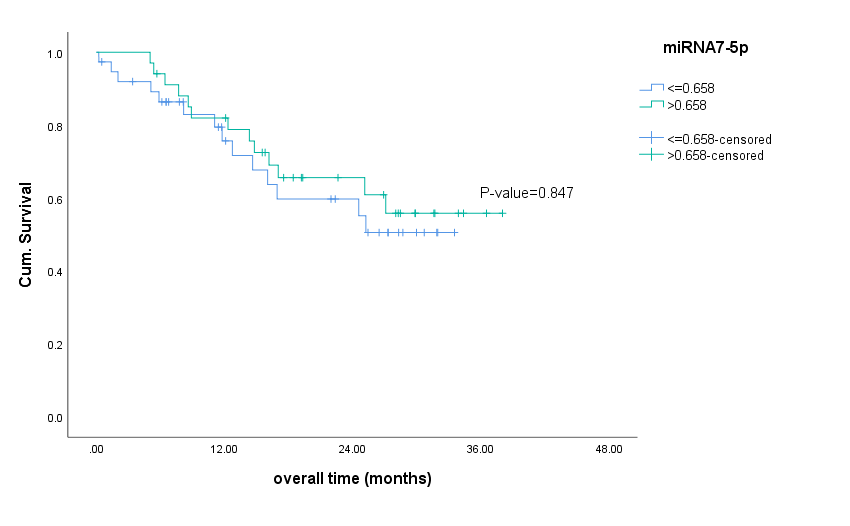


**Figure S6.** Effect of miRNA7-5p on the OS of 77 CRC patients before 5-FU therapy.


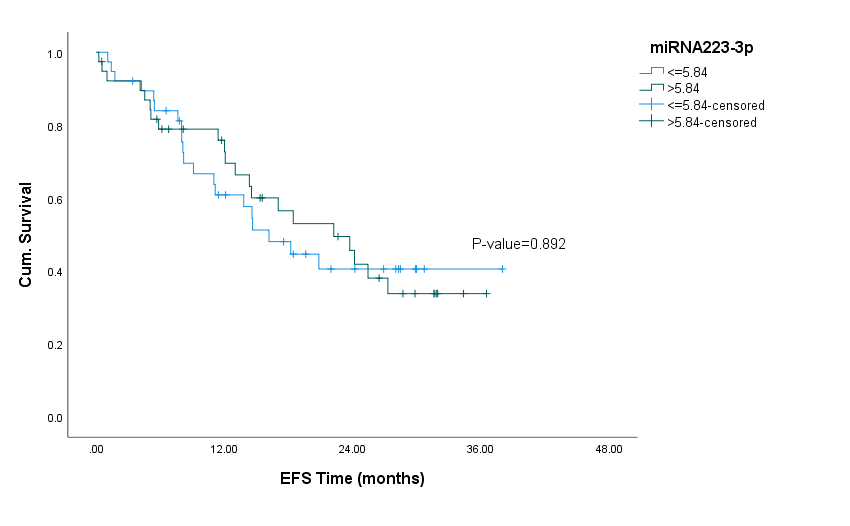


**Figure S7.** Effect of miRNA223-3p on the EFS of 77 CRC patients before 5-FU therapy.


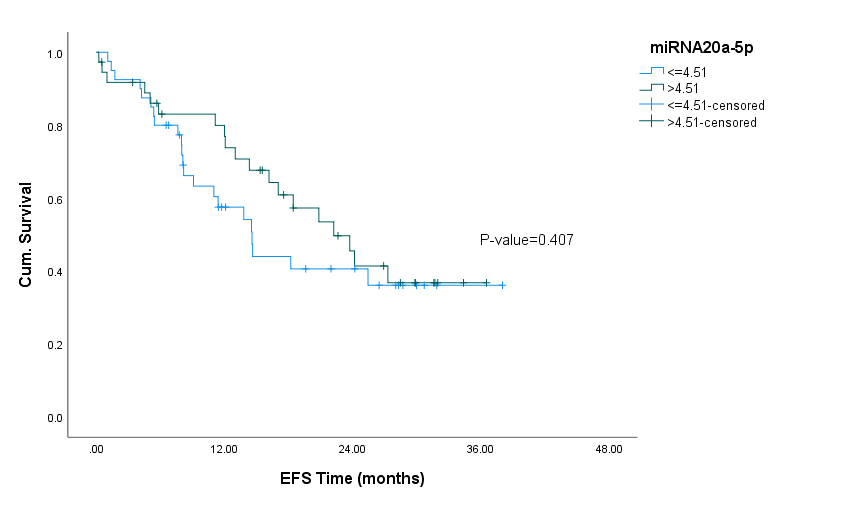

**Figure S8.** Effect of miRNA20a-5p on the EFS of 77 CRC patients before 5-FU therapy.


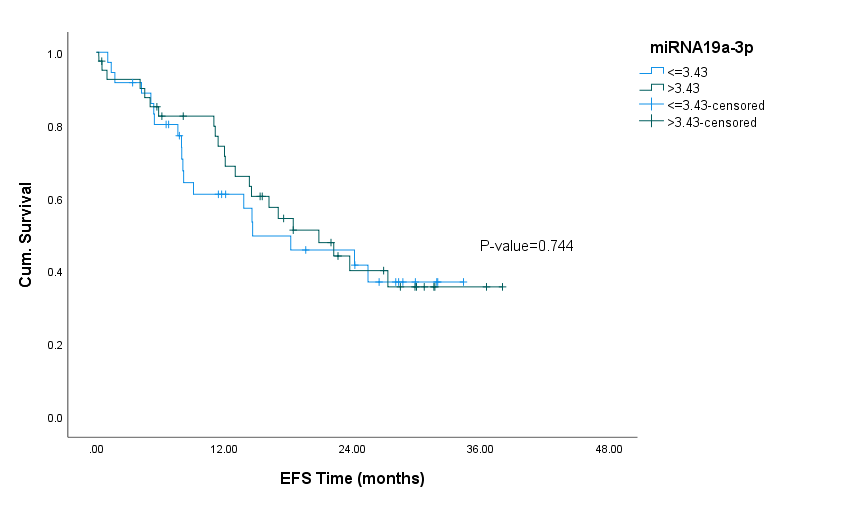


**Figure S9.** Effect of miRNA19a-3p on the EFS of 77 CRC patients before 5-FU therapy.


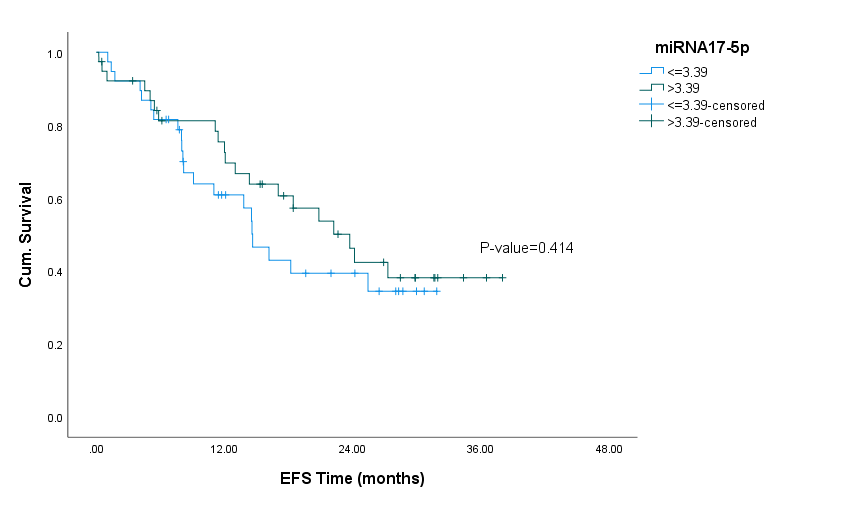


**Figure S10.** Effect of miRNA17-5p on the EFS of 77 CRC patients before 5-FU therapy.


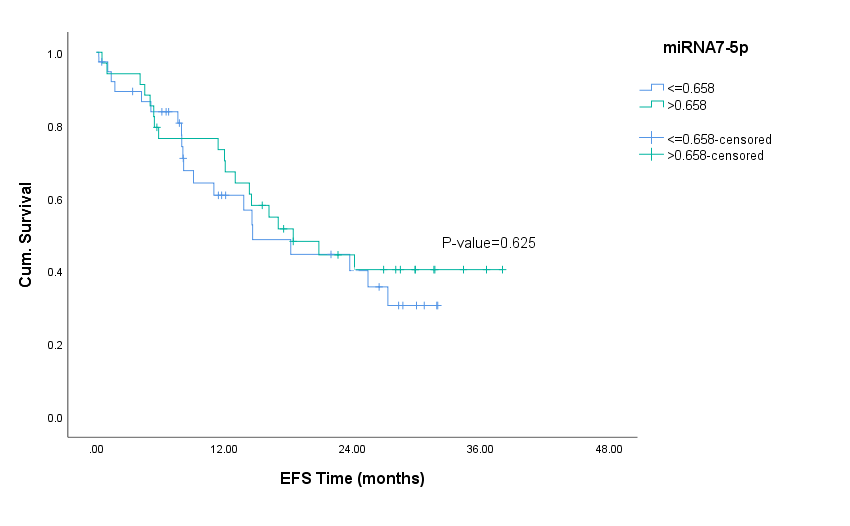


**Figure S11.** Effect of miRNA7-5p on the EFS of 77 CRC patients before 5-FU therapy.
